# Biosynthesis of coelulatin for the methylation of anthraquinone featuring HemN-like radical S-adenosyl-L-methionine enzyme

**DOI:** 10.3389/fmicb.2022.1040900

**Published:** 2022-11-17

**Authors:** Lishuang Nie, Tianyi Wei, Mingming Cao, Yunbin Lyu, Shaochen Wang, Zhiyang Feng

**Affiliations:** ^1^College of Food Science and Technology, Nanjing Agricultural University, Nanjing, China; ^2^State Key Laboratory of Bio-Organic and Natural Products Chemistry, Shanghai Institute of Organic Chemistry, Chinese Academy of Sciences, Shanghai, China

**Keywords:** soil metagenome, aromatic polyketides, type II polyketide synthase, heterologous expression, radical SAM enzyme

## Abstract

Bacterial aromatic polyketides are usually biosynthesized by the type II polyketide synthase (PKS-II) system. Advances in deoxyribonucleic acid (DNA) sequencing, informatics, and biotechnologies have broadened opportunities for the discovery of aromatic polyketides. Meanwhile, metagenomics is a biotechnology that has been considered as a promising approach for the discovery of novel natural products from uncultured bacteria. Here, we cloned a type II polyketide biosynthetic gene cluster (BGC) from the soil metagenome, and the heterologous expression of this gene cluster in *Streptomyces coelicolor* M1146 resulted in the production of three anthraquinones, two of which (coelulatins **2** and **3**) had special hydroxymethyl and methyloxymethyl modifications at C2 of the polyketide scaffold. Gene deletion and *in vitro* biochemical characterization indicated that the HemN-like radical S-adenosyl-L-methionine (SAM) enzyme CoeI exhibits methylation and is involved in C2 modification.

## Introduction

Bacterial aromatic polyketides are a structurally diverse class of natural products with various bioactivities and are usually biosynthesized by the type II polyketide Synthase (PKS-II) system encoded by the biosynthetic gene cluster (BGC) in the bacterial genome. Many aromatic polyketides have been isolated from bacteria, and their biosynthetic gene cluster in the bacterial genome have also been cloned and characterized. However, because most bacteria cannot be cultured under current laboratory conditions, the discovery of both novel compounds and biosynthetic genes is limited (Ryan et al., 2009). It is well established that environmental samples contain significantly greater bacterial diversity than cultured samples. Libraries of deoxyribonucleic acid (DNA) extracted directly from environmental samples provide a means to access natural products and their biosynthetic genes in the genomes of previously inaccessible bacteria (Ryan et al., 2009). This approach, which is termed “metagenomics,” provides an alternative methodology to identify novel bioactive natural products.

The complexity and diversity of aromatic polyketides with new bioactivities can be supported by regulatory reactions. Understanding and engineering modification processes help to derive different new aromatic polyketides through a rational combination of regulatory reactions (Wang et al., 2020). Radical S-adenosyl-L-methionine (SAM) enzymes are a group of such proteins involved in regulatory modifications of natural products. The radical SAM superfamily is unified by the presence of a unique threecysteine motif (most often CxxxCxxC) that binds a [4Fe−4S] cluster (Holliday et al., 2018; Sinner et al., 2022). These enzymes usually utilize a [4Fe−4S] cluster and SAM to initiate a diverse set of radical reactions, in most or all cases, *via* the formation of a 5′-deoxyadenosyl radical (dAdo•) intermediate. Radical SAM enzymes are responsible for a wide range of reactions, including the formation of protein-free radicals, methylation, sulfur insertion, methylthiolation, oxidation, isomerization, and cleavage of C–C bonds using radical chemistry (Frey et al., 2008; Buckel and Thauer, 2011; Zhang et al., 2012; Broderick et al., 2014; Mehta et al., 2015). Recent studies showed that radical SAM enzyme-catalyzed reactions are more diverse and complex than initially anticipated. Mechanistic studies of these reactions revealed unprecedented free radical chemistry and precisely controlled reaction pathways, which dramatically changed our view of enzymology and chemistry (Ruszczycky et al., 2018; Broderick and Broderick, 2019).

HemN belongs to the radical SAM superfamily, which catalyzes the anaerobic oxidative decarboxylation of coproporphyrinogen III to form protoporphyrinogen IX in the biosynthesis of heme (Cheng et al., 2022). HemN-like enzymes share a high sequence homology with HemN and may bind two SAM molecules simultaneously by acting on methylated carbon centers (Fujimori, 2013; Bauerle et al., 2015). This enzyme subfamily is involved in the biosynthesis of various natural products with different types of reactions (Cheng et al., 2022). Huang et al. (2012) revealed that YtkT, the first HemN-like enzyme characterized *in vitro*, is essential for the formation of the cyclopropyl moiety in the biosynthesis of the natural product yatakemycin. In 2014, it was confirmed that Jaw5 catalyzed the formation of the polycyclopropanated backbone during the biosynthesis of jawsamycin (FR-900848) *in vivo* (Hiratsuka et al., 2014). Mahanta et al. (2017) proved that TbtI was responsible for catalyzing the methylation of a thiazole moiety in the biosynthesis of thiomuracin. Research on HemN-like enzymes has expanded in recent years, and these enzymes appear to be much more diverse than originally anticipated. Further studies on the structure, molecular basis, and biological functions of these enzymes are likely to drive significant advances in enzyme engineering and future applications in a wide range of biosynthesis.

In this study, we identified a type II polyketide BGC containing a radical SAM gene in a cosmid clone (YN1903) using a metagenomic approach. The cosmid YN1903 was then introduced into *Streptomyces coelicolor* M1146 by intergeneric conjugation. Polyketide compounds from the *S. coelicolor* M1146 conjugant were identified, and the radical SAM enzyme CoeI, which plays a key role in regulating modifications of polyketide biosynthesis, was characterized.

## Materials and methods

### Bacterial strains, plasmids, and media

*Streptomyces coelicolor* M1146 was used as a host for the heterologous expression of the type II gene cluster. *Escherichia coli* EPI100 and the pTG19-T vector were used for general cloning. *E. coli* JTU007/pUZ8002 and the pOJ436 vector were used for the conjugation of *E. coli*/*Streptomyces*. *E. coli* BW25113/pKD47 was used for gene knockout. The pET-30a vector and *E. coli* BL21 were used for protein expression. Biochemicals and media were purchased from Sangon Biotech Co., Ltd. (Shanghai, China) and Wanqing Co., Ltd. (Nangjing, China) unless otherwise stated. Restriction enzymes were purchased from TaKaRa Biotechnology Co., Ltd. (Beijing, China).

### Library screening and sequence analysis

Cosmid DNA was isolated from the Yunnan soil library as the template to amplify the KS_α_ sequence using the 540F&1100R primer, which was designed based on the KS_α_ domain in type II polyketide synthase (Wawrik et al., 2005; Wang et al., 2017). Amplicons of the correct predicted size [560 base pair (bp)] were gel-purified, sequenced, and compared with deposited KS_α_ genes in the National Center for Biotechnology Information (NCBI) database. Unique KS_α_ genes were used as probes to recover type II PKS-containing clones by a serial dilution method using the following touchdown protocol: denaturation (95°C, 4 min), 10 touchdown cycles (95°C, 40 s; 65°C [−1°C per cycle up to 55°C], 40 s; 72°C, 1 min); 30 standard cycles (95°C, 40 s; 55°C, 40 s; and 72°C, 40 s); and a final extension step (72°C, 10 min).

Open reading frames (ORFs) were deduced from the sequence with the assistance of the RAST server program (https://rast.nmpdr.org/). The corresponding deduced proteins were compared with other known proteins in the databases using available BLAST methods (http://www.ncbi.nlm.nih.gov/blast/).

### Compound fermentation and analysis

Cosmid YN1903 was retrofitted with the oriT- and Amp^R^-containing *Dra* I fragment from pOJ436 (Bierman et al., 1992). Retrofitted cosmid was then conjugated from *E. coli* JTU007/PUZ8002 into *S. coelicolor* M1146 for the heterologous expression *via* a standard intergeneric conjugation protocol (Feng et al., 2011; Musiol et al., 2011).

The seed culture of the *S. coelicolor* YN1903 strain was prepared by inoculating 0.5 ml of the spore with 50 ml of R5 liquid medium, and the resultant solution was incubated for 3 days at 28°C with shaking at 225 revolutions per minute (rpm). Then, the seed culture was inoculated in 50 ml of ISP4 liquid medium (1:100), to which 5 g of HP-20 resin was added. The cultures were incubated for 7 days at 28°C with shaking at 225 rpm. After fermentation, HP-20 resin was rinsed with water and then dried by air. Next, the resin was extracted three times with 100% methanol. Methanol extracts were combined, concentrated, and subjected to high-performance liquid chromatography (HPLC) analysis. Methanol eluents were analyzed by HPLC (1 ml/min) using a linear gradient from 80:20 H_2_O:MeOH to 100% MeOH over 40 min.

### The isolation and analysis of metabolites

Metabolites obtained in the methanol extraction were subjected to silica gel column chromatography for the first-round isolation. The crude extract was subjected to a silica gel column by elution with a mixture of CH_2_Cl_2_ and MeOH, with a gradient from 100:1 → 50:1 → 20:1 → 10:1 → 5:1, and the elution was detected by HPLC. The extract was further purified by semi-preparative HPLC separation (Fisher Wharton C18, 5 μm, 10 mm × 250 mm) on Shimadzu LC-20A. The column was equilibrated with 40% solvent A (H_2_O containing 0.1% formic acid)/60% solvent B (MeOH) and developed with the following program: 0 −30 min, a linear gradient increase from 40% A/60%B to 100%B. The flow rate was 4 ml/min, and the detection wavelength was 435 nm.

For the detection of high-resolution mass spectrometry (HR-MS), the extracts were dissolved in chromatographic-grade methanol and centrifuged for 10 min. The resultant clear supernatant (10 μl) was used for MS analysis. HR-MS was performed on Agilent Q-TOF 6520A. A nuclear magnetic resonance (NMR) study was performed on a 600-MHz NMR (BRUKER AVANCE III 600). Data were generated for triplicate experiments.

### Gene deletion of YN1903

Polymerase chain reaction- (PCR-) targeting system was used for in-frame deletion of the genes in the YN1903 gene cluster (Gust et al., 2002). The primers used in this study are listed in Supplementary Table S1; all primers used for gene deletion contained a *Bcu* I-recognized site at their 5′ end. Using apramycin-resistance gene as the template, Apr fragments with *Bcu* I cleavage sites on both sides were amplified. Then, 39-bp bases were selected from the upstream and downstream regions of the target gene as the homologous arm, and the amplified fragment in the above step was used as the template to amplify the apramycin-resistance gene fragment with homologous arms. The PCR amplification protocol was as follows: denaturation at 95°C for 3 min, 5 touchdown cycles at 98°C for 1 min; 68°C [−2°C per cycle until 58°C was reached] for 15 s; and 72°C for1 min 30 s); 30 standard cycles at 98°C for 1 min; 58°C for 15 s; and 72°C for 1 min 30 s; and a final extension step at 72°C for 10 min. The obtained gene fragment was then electrically transferred into the competent cell *E. coli* BW25113/pKD47/YN1903. The recombinant plasmid was digested by the *Bcu* I enzyme and then ligated with the T4 DNA ligase to obtain the plasmid knockout of the target gene. The mutant plasmid was confirmed by sequence analysis and was subsequently conjugated into *S. coelicolor* M1146 for fermentation.

### Construction of protein overexpressing strain

Deoxyribonucleic acid (DNA) was extracted from cosmid YN1903 as the template, and a DNA fragment (amplified with the primers *coeI* protein-F and *coeI* protein-R) containing the *coeI* gene was cloned into the pTG19-T vector; after verification by sequencing, the 1.48-kb *Nde* I/*Xhol* I fragment was recovered from the pTG19-T vector and then ligated into the same site of pET-30a to yield the pET*coeI* plasmid, which was then introduced into the bacterial expression strain *E. coli* BL21 to obtain *E. coli* BL21/pET*coeI* for expressing CoeI to give a C-terminal 6 × His-tagged protein.

### Purification of CoeI

*Escherichia coli* BL21/pET*coeI* was inoculated into 4 ml of LB medium containing 50 μg/ml of kanamycin. The culture was grown at 37°C overnight and then transferred to 400 ml of Lysogeny Broth (LB) medium containing 50 μg/ml of kanamycin and 0.25 mM of ammonium ferrous sulfate. When OD_600_ of the culture reached ~0.3, 50 μl of 0.3 M cysteine and 100 μl of 0.1 M ammonium ferrous sulfate were added, and the culture was grown at 37°C until an OD_600_ of ~0.6 was reached. Then, 50 μl of 1 M isopropyl β-d-1-thiogalactopyranoside (IPTG) was added, and the culture was incubated at 18°C for another 20 h.

The cultures were harvested by centrifugation at 4,000 × *g* for 10 min. The collected cells were resuspended in 10 ml of lysis buffer (NaH_2_PO_4_ 300 mM, NaCl 50 mM, imidazole 10 mM, and pH 8.0) containing 1 mg/ml of lysozyme, and the resuspended cells were disrupted by ultrasonication. The bacterial suspension was centrifuged at 4,000 × *g* for 30 min. The supernatant was then subjected to affinity purification on a column by elution with different concentrations of imidazole prepared by mixing lysis buffer with elution buffer. The protein was eluted in 3 ml of 250 mM imidazole elution buffer, subjected to a desalination column to eliminate salt ions, and finally dissolved in a 3-ml Tris•HCl buffer (Tris 50 mM, NaCl 100 mM, glycerol 10%, and pH 8.0). The purified protein was confirmed on a 12% sodium dodecyl sulfate-polyacrylamide gel electrophoresis (SDS-PAGE) gel. The obtained protein was concentrated again to 200 μl using a 50-kDa ultrafiltration tube, snap-frozen in liquid nitrogen, and stored at −80°C until further use.

### Reconstitution of CoeI

The radical SAM enzyme needs to be reconstituted under anaerobic conditions before enzymatic assays. Reconstitution was performed at 4°C (Jin et al., 2018). Approximately 30 μl of 1 M dithiothreitol (DTT) was added to 3 ml of protein dissolved in Tris•HCl buffer, and the mixture was inoculated for 15 min. Then, 30 μl of 50 mM ammonium ferrous sulfate was added to make a final concentration of 0.5 mM; then, after 45 min, 10 μl of 50 mM sodium sulfide was added every 30 min for three times to make a final concentration of 0.5 mM and reconstituted for at least 3 h. Finally, the reconstituted mixture was treated on a desalination column to obtain the dark-brown protein in 3-ml Tris•HCl buffer.

### Computational docking experiment of CoeI, the substrate, and cofactors

Computational docking experiment was performed using Alphafold v2.4.

### Anaerobic treatment of solutions

All solutions used in an anaerobic glove box required prior anaerobic treatment. The solution was placed in a flask and immersed in liquid nitrogen for snap-freezing. The completely frozen solution in the flask was evacuated in a vacuum and then placed in flowing water. This process was repeated three times. After replacing oxygen inside the flask three times by withdrawing nitrogen, the flask containing the solution was placed in an anaerobic glove box.

### *In vivo* and *in vitro* enzymatic assays

*In vivo* enzymatic assay was tested by whole-cell transformation experiments. Whole-cell transformation experiments were performed as follows: 400 ml of cultured cells of *E. coli* BL21/pET*coeI* for protein expression were centrifuged and washed two times with pre-chilled phosphate-buffered saline (PBS) buffer (pH 7.0). The resuspended cell pellet was then lysed in 40 ml of PBS buffer; 10 ml of the suspension was transferred to a 50-ml centrifuge tube; and 10-mM substrate was added. The reactions were incubated at 30°C, 225 rpm, for 12 h.

All *in vitro* enzymatic assays were performed in an anaerobic glove box with <1 parts per million (ppm) of O_2_. Enzymatic reactions were conducted in Tris•HCl buffer (Tris 50 mM, NaCl 100 mM, glycerol 10%, pH 8.0) with the following components: 1 μM reconstituted CoeI, 10 μM substrate, 1 mM SAM, 5 mM DTT, 5 mM MgCl_2_, 5 mM Na_2_S_2_O_4_, and 7% dimethyl sulfide (DMSO). The reactions were incubated at 30°C for 12 h and then quenched with methanol. Data from triplicate experiments were collected.

### Liquid chromatography–mass spectrometry and HPLC analyses of enzymatic products

Liquid chromatography–mass spectrometry (LC–MS) analysis of enzymatic products was performed in a negative ion mode by a reverse-phase column (Grace Alltech Alltima, C18, 5 μm, 100 Å, 10 × 250 mm) on an Agilent 1200 series. The gradient elution was as follows: 0–29 min, a linear increase from 50% A (H_2_O)/50% B (MeOH) to 5% A/95% B; 29–31.5 min, 5% A/95% B; and 31.5–34 min, a linear increase to 50% A/50% B. The flow rate was 0.8 ml/min, and the detection wavelength was 435 nm.

High-performance liquid chromatography analysis of enzymatic products was performed using a reverse-phase column (Grace Alltima, C18, 5 μm, 100 Å, 10 mm × 250 mm) on an Agilent 1200 series. The gradient elution was as follows: 0–30 min, a linear increase from 40% A (H_2_O)/60% B (MeOH) to 0% A/100% B and 30–35 min, 0% A/100% B. The flow rate was 0.8 ml/min, and the detection wavelength was 435 nm. Data from triplicate experiments were collected.

### GenBank accession numbers

The sequence data of the genes in the COE gene cluster were deposited in GenBank under accession nos. MN601984–MN601997.

## Results

### The identification and heterologous expression of COE BGC

**Figure 1:**
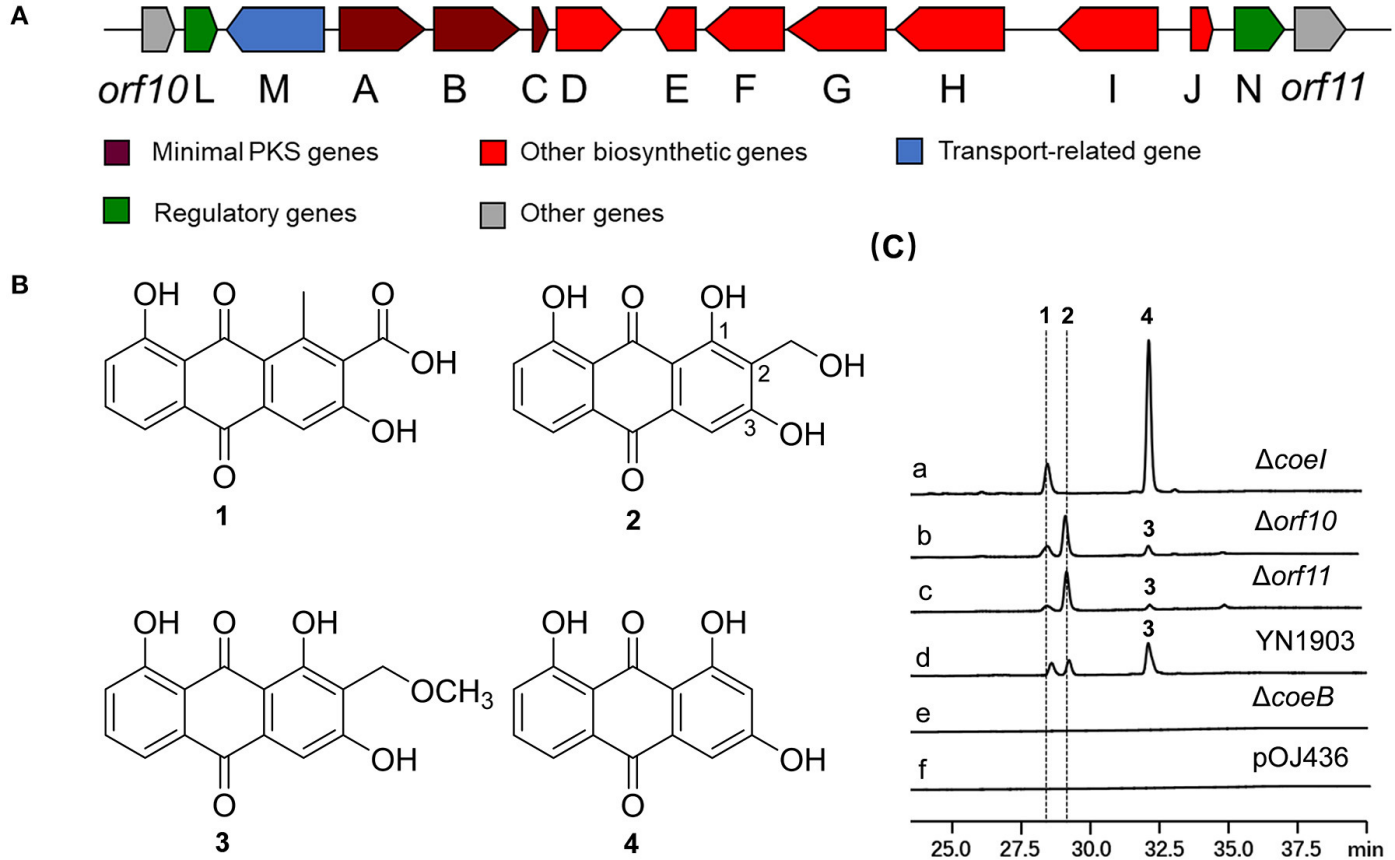
**(A)** Organization of the COE biosynthetic gene cluster (BGC) in YN1903. **(B)** Chemical structures of compounds **1**, **2**, **3**, and **4**. **(C)** High-performance liquid chromatography (HPLC) analysis of relevant metabolites (ultraviolet (UV) at 435 nm). **(a)** *Streptomyces coelicolor* YN1903Δ*coeI, S. coelicolor* M1146 harboring *coeI* deleted the COE cluster; **(b)** *S. coelicolor* YN1903Δ*orf10, S. coelicolor* M1146 harboring *orf10* deleted the COE cluster; **(c)** *S. coelicolor* YN1903Δ*orf11, S. coelicolor* M1146 harboring *orf11* deleted the COE cluster; **(d)** *S. coelicolor* YN1903, *S. coelicolor* M1146 harboring the COE cluster; **(e)** *S. coelicolor* YN1903Δ*coeB, S. coelicolor* M1146 harboring *coeB* deleted the COE cluster; and **(f)** *S. coelicolor* M1146 harboring the vector pOJ436.

A cosmid clone (YN1903) with a 42-kb insert containing PKS-II genes was identified by PCR from a soil metagenomic library (Wang et al., 2017). The cosmid DNA was retrofitted with the *Dra* I fragment-containing *oriT* and Amp^R^ from pOJ436 and then conjugated into *S. coelicolor* M1146. An HPLC analysis of the fermentation broth of *S. coelicolor* YN1903 (*S. coelicolor* M1146 harboring the COE cluster) showed that three clone special compounds were produced [Figure 1C(d)]. These compounds were purified, and their structures were elucidated by HR-MS and NMR spectroscopy. HR-MS revealed that compound **1** has the chemical formula C_16_H_10_O_6_ ([M–H]^−^ 297.0406, observed, 297.0399, calculated). The ^13^C NMR data (Supplementary Figure S13 and Supplementary Table S2) of compound **1** showed 16 carbon signals (δ_C_ 142.3, 122.4, 161.2, 112.9, 136.4, 118.6, 136.7, 124.8, 161.9, 117.3, 189.7, 130.7, 182.5, 132.9, 20.43, and 168.8), which were consistent with the published data (Krupa et al., 1989). The integral value of a single peak (δ_H_ 2.51) in ^1^H NMR of compound **1** is 3, which represents the methyl proton in the structure and belongs to the 1-CH_3_ group. In the heteronuclear multiple bond correlation (HMBC) spectra, the hydroxyl groups at the C-3 and C-8 positions had a strong coupling with the quaternary carbon of δ_C_ 161.2 and δ_C_ 161.9, respectively, so δ_C_ 161.2 was assigned to C-3 and δ_C_ 161.9 was assigned to C-8. A carboxyl group (δ_C_ 168.8) belonging to 2-COOH was supported by HMBC correlation with H_3_-1–CH_3_. Therefore, compound **1** was identified as 3,8-dihydroxy-1-methylanthraquinone-2-carboxylic acid (DMAC), a polyketide intermediate that was isolated from an engineered *Streptomyces* strain with a known biosynthetic pathway in that strain (Javidpour et al., 2013) (Supplementary Figures S12–S16 and Supplementary Table S2). The HR-MS analysis of compound **2** gave the chemical formula C_15_H_10_O_6_ ([M–H]^−^ 285.0406, observed, 285.0399, calculated). TheHR-MS analysis of compound **3** yielded the chemical formula C_16_H_12_O_6_ ([M–H]^−^ 299.0563, observed, 299.0556, calculated). A careful comparison of the NMR data (Supplementary Figures S12–S23 and Supplementary Table S2) demonstrated that the structures of **2**, **3** were highly similar to those of compound **1**. Unlike compound **1**, there was a hydroxyl group at the C-1 position of compound **2** (δ_C_ 165, OH-1), and a hydroxymethyl group at the C-2 position (δ_C_/δ_H_ 51.6/4.50, CH_2_OH-2). The only difference between compounds **3** and **2** was that there was a methyloxymethyl group at the C-2 position of compound **3** (δ_C_/δ_H_ 58.1/4.43; δ_C_/δ_H_ 61.6/2.61, CH_2_OCH_3_-2). The interpretation of NMR data revealed that compounds **2** and **3** were coelulatins A and B, respectively, which had previously been reported as plant metabolites (Bowie et al., 1962) (Figure 1B). Interestingly, the hydroxymethyl and methyloxymethyl groups at C-2 of compounds **2** and **3** are distinguished from the reported bacterial aromatic polyketides, implying a rare modification step at C-2 during the biosynthesis of coelulatin.

### *In silico* analysis and identification of biosynthetic genes in the COE gene cluster

**Figure 2:**
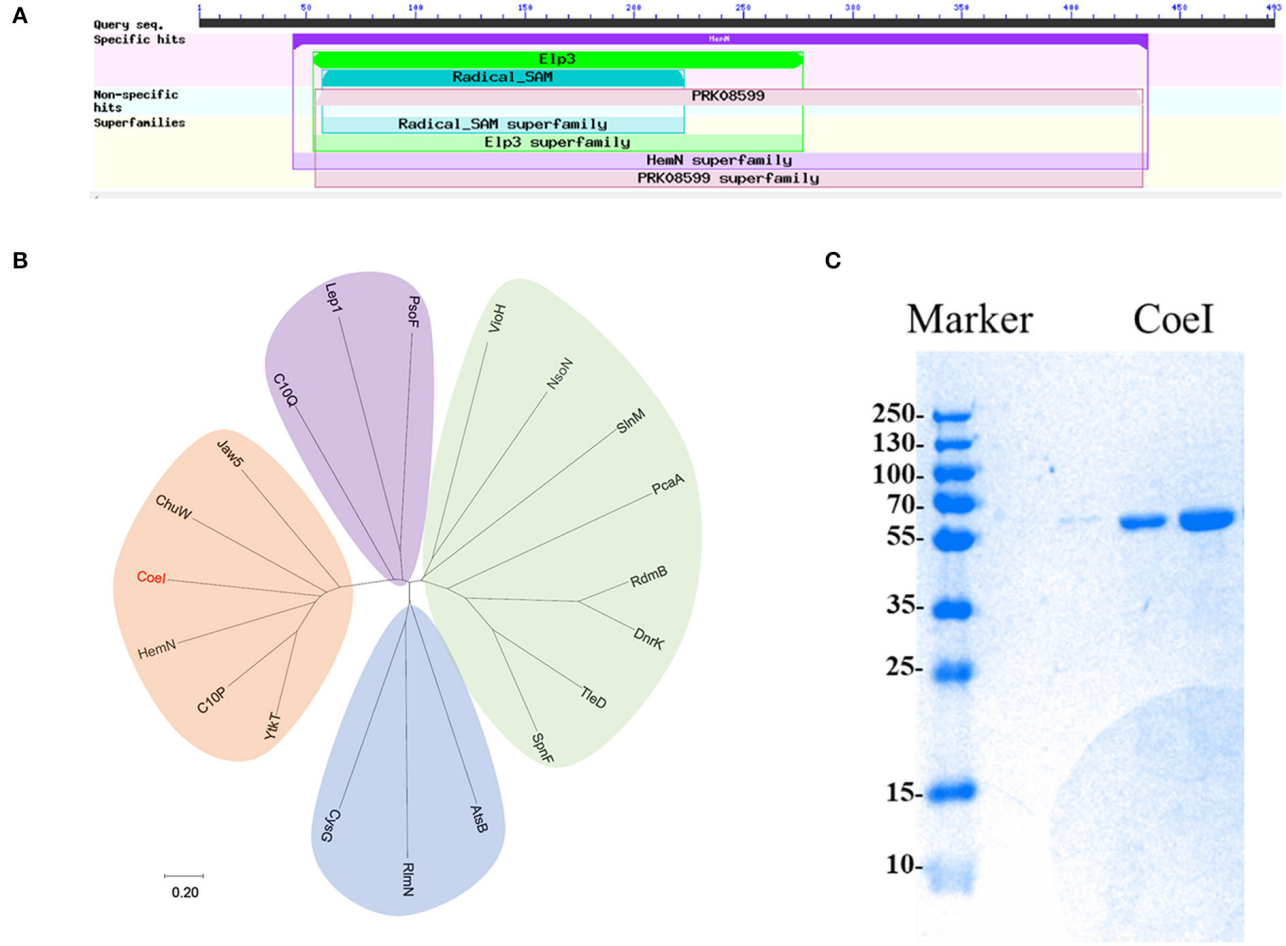
**(A)** The predicted domain analysis of CoeI in the National Center for Biotechnology Information (NCBI) database. **(B)** The phylogenetic analysis of CoeI with different radical S-adenosyl-L-methionine (SAM) enzymes. Functional known radical SAM enzymes were selected from different bacterial strains. CoeI and 5 HemN-like enzymes were cladded together in the phylogenetic tree. **(C)** The analysis of purified recombinant CoeI by sodium dodecyl sulfate-polyacrylamide gel electrophoresis (SDS-PAGE).

In-frame-deletion was performed to elucidate the role of various genes in biosynthesis. The gene *coeB* in-frame deletion mutant strain *S. coelicolor* YN1903ΔcoeB failed to produce polyketide compounds, supporting a corresponding relationship between polyketide compounds and COE BGC [Figure 1C(e)]. In-frame-deletions of *orf10* and *orf11* did not affect the production of metabolites, indicating the boundaries of the BGC [Figure 1C(b,c)]. The genes between *orf10* and *orf11* were analyzed, and their homologies and deduced functions are listed in Table 1. The COE gene cluster contained 13 genes, including polyketide skeleton biosynthetic genes, regulating modification genes, and transcription regulation genes (Table 1). Bioinformatic analysis revealed that the CoeI protein belongs to the family of HemN-like enzymes (Figure 2A). Phylogenetic analysis revealed that CoeI was clustered with homologs in a HemN-like escalade separated from the other radical SAM enzymes (Figure 2B). All five homologs belonged to HemN-like radical SAM enzymes and catalyzed a variety of reactions, including decarboxylation (Layer et al., 2003), hydroxylation (Jansson et al., 2003), cyclization (Layer et al., 2002; Wu et al., 2017), ring opening reaction (LaMattina et al., 2016), and cyclopropanation (Hiratsuka et al., 2014).

**Table 1:** Homologous analysis of deduced proteins in the COE gene cluster.

**Protein**	**aa**	**The most homologous proteins, their ID, and source**	**Identity %**
ORF10	370	WP_016828267.1. Integrase core domain-containing protein, *S. viridosporus*	85
CoeL	161	WP_123976907.1. Transcriptional regulator, MarR family, *Streptomyces* sp. Ag109 O5-1	79
CoeM	484	WP_091283396.1. MFS transporter permease subunit, *Frankia*	70
CoeA	423	WP_175482876.1. Polyketide beta-ketoacyl synthase, *A. iranica*	82
CoeB	417	WP_091451475.1. Polyketide chain length factor, *A. iranica*	74
CoeC	76	WP_018540030.1. Acyl carrier protein, unclassified *Streptomyces*	52
CoeD	306	NYF59551.1. Cyclase, *M. purpureochromogenes*	65
CoeE	249	RBL80413.1. Ketoreductase, *S. cavourensis*	46
CoeF	384	WP_059205885.1. Aminopeptidase, *S. canus*	61
CoeG	487	WP_169347312.1. Aromatase/cyclase, *Pyxidicoccusfallax*	32
CoeH	537	WP_009739948.1. Long-chain-fatty-acid-CoA ligase, *Frankia* sp. QA3	56
CoeI	493	MPQ97192.1. Radical SAM enzyme, *M.deserti*	82
CoeJ	261	RLU89239.1. Ketoreductase, *S. griseocarneus*	74
CoeN	249	SDD12127.1. Regulatory protein, *A. iranica*	57
ORF11	267	MYX43475.1. DNA-3-methyladenine glycosylase II, *Streptomyces* sp. SID89	73

To determine the function of CoeI in the biosynthesis of YN1903 compounds, the *coeI* in-frame-deletion mutant *S. coelicolor* YN1903Δ*coeI* was constructed. In contrast with the strain *S. coelicolor* YN1903, *S. coelicolor* YN1903Δ*coeI* did not produce metabolites **2** and **3**; however, it accumulated an intermediate product **4** [Figure 1C(a)]. High-resolution electrospray ionization mass spectrometry (HR-ESI-MS) analysis of the intermediate product **4** revealed the chemical formula C_15_H_10_O_6_ ([M–H]^−^ 255.0303, observed, 255.0293, calculated). The interpretation of NMR data (Supplementary Figures S24–S28 and Supplementary Table S3) revealed that the intermediate product **4** was the C-2 de-hydroxymethyl form of **2** (Figure 1B). Thus, it was confirmed that CoeI played a critical role in C-2 modification in the biosynthesis of **2** and **3**.

### Expression, purification, and reconstitution of the protein CoeI

The ORF of *coeI* was amplified by PCR, and the PCR product was ligated with the pET-30a vector to obtain pET*coeI*. Plasmid pET*coeI* was transferred into *E. coli* BL21, and the transformant was cultured for CoeI expression. The recombinant CoeI protein was purified using a Ni-NTA column and detected by SDS-PAGE. A clone special band of 56.7 kDa when analyzing the supernatant of the broken cell on the SDS-PAGE corresponded to the predicted size of the recombinant CoeI well (Figure 2C). The concentration of purified recombinant CoeI was 20.16 mg/ml.

To reconstitute the tetrairon–tetrasulfur cluster necessary for the use of ferrous ammonium sulfate and sodium sulfide in the catalysis of HemN-like radical SAM enzymes, CoeI was reconstituted before the *in vitro* analysis in an anaerobic glove box (Jin et al., 2018). The CoeI protein exhibited a light brown color before reconstitution, but a brown–black color after reconstitution, which was identical to the color of a HemN-like SAM enzyme reported previously (Jin et al., 2018).

### Computational docking experiment of CoeI, the substrate, and cofactors

**Figure 3:**
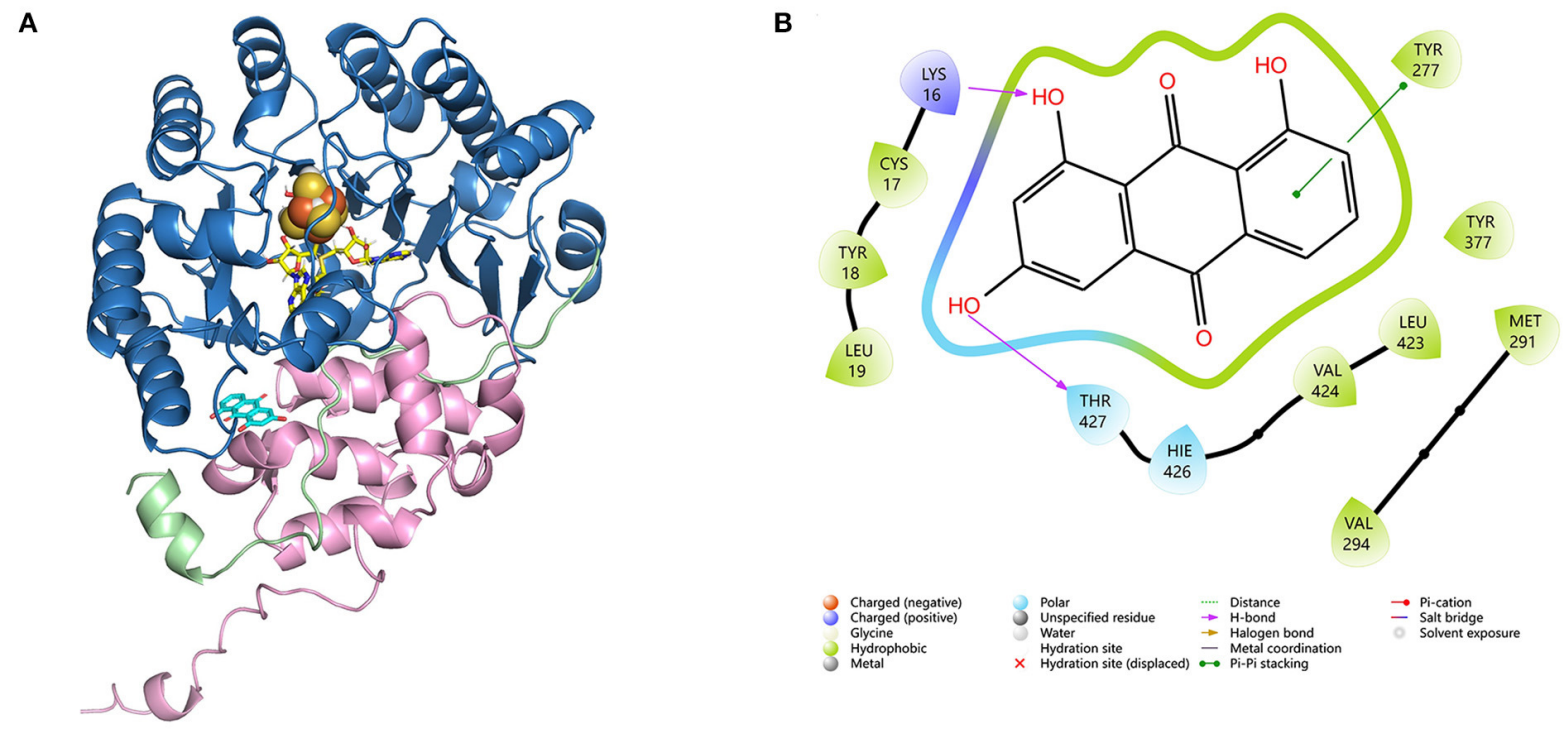
**(A)** Molecular docking of CoeI, the substrate, and cofactors. CoeI consists of two distinct domains (shades of blue and pink), as well as an elongated N-terminal region, which is termed a trip-wire (green). The N-terminal region bears a barrel, which binds all cofactors, a 4Fe-4S cluster (orange), and two SAM molecules (yellow). The N-terminal trip-wire and the C-terminal domain probably participate in substrate (cyan) binding. **(B)** An interaction between compound 4 and the enzyme binding site in CoeI. Computational docking was carried out using Alphafold v2.4.

HemN-like enzymes contain a unique three-cysteine motif (CxxxCxxC) (Supplementary Figure S1) that binds a [4Fe−4S] cluster, which acts as a direct initiator of the enzyme reaction. These proteins also contain two SAM binding sites (Supplementary Figure S1). To understand how CoeI recognizes its substrate, computational docking was performed using a substrate (compound **4**), a [4Fe−4S] cluster, and two SAM molecules as a ligand. The structure of CoeI is very similar to that of HemN (Layer et al., 2003). CoeI consists of two distinct domains. The N-terminal region bears a barrel that binds all cofactors, a 4Fe−4S cluster, and two SAM molecules. The N-terminal trip-wire and the C-terminal domain are probably involved in substrate binding (Figure 3A). The substrate (compound **4**) hydroxyl is linked by the side chains of Lys16 and Thr427 in CoeI with an H-bond (Figure 3B).

### *In vitro* and *in vivo* assays of the protein CoeI

Whole-cell transformation experiments were performed using proteins extracted from *E. coli* BL21/pET*coeI* culture. When product **4** was used as a feedstock, in addition to products **2** and **3**, a new product **5** was also detected (Supplementary Figure S3). The HR-MS analysis of product **5** identified the chemical formula C_15_H_10_O_5_ ([M–H]^−^ 269.0460, observed, 269.2320, calculated). The interpretation of NMR data (Supplementary Figures S29–S33 and Supplementary Table S3) revealed that product **5** was a C-2 methylated form of product **4** (Supplementary Figure S6). Further, product **5** was also introduced into whole-cell transformation but could not be transformed into any product (Supplementary Figure S3).

**Figure 4:**
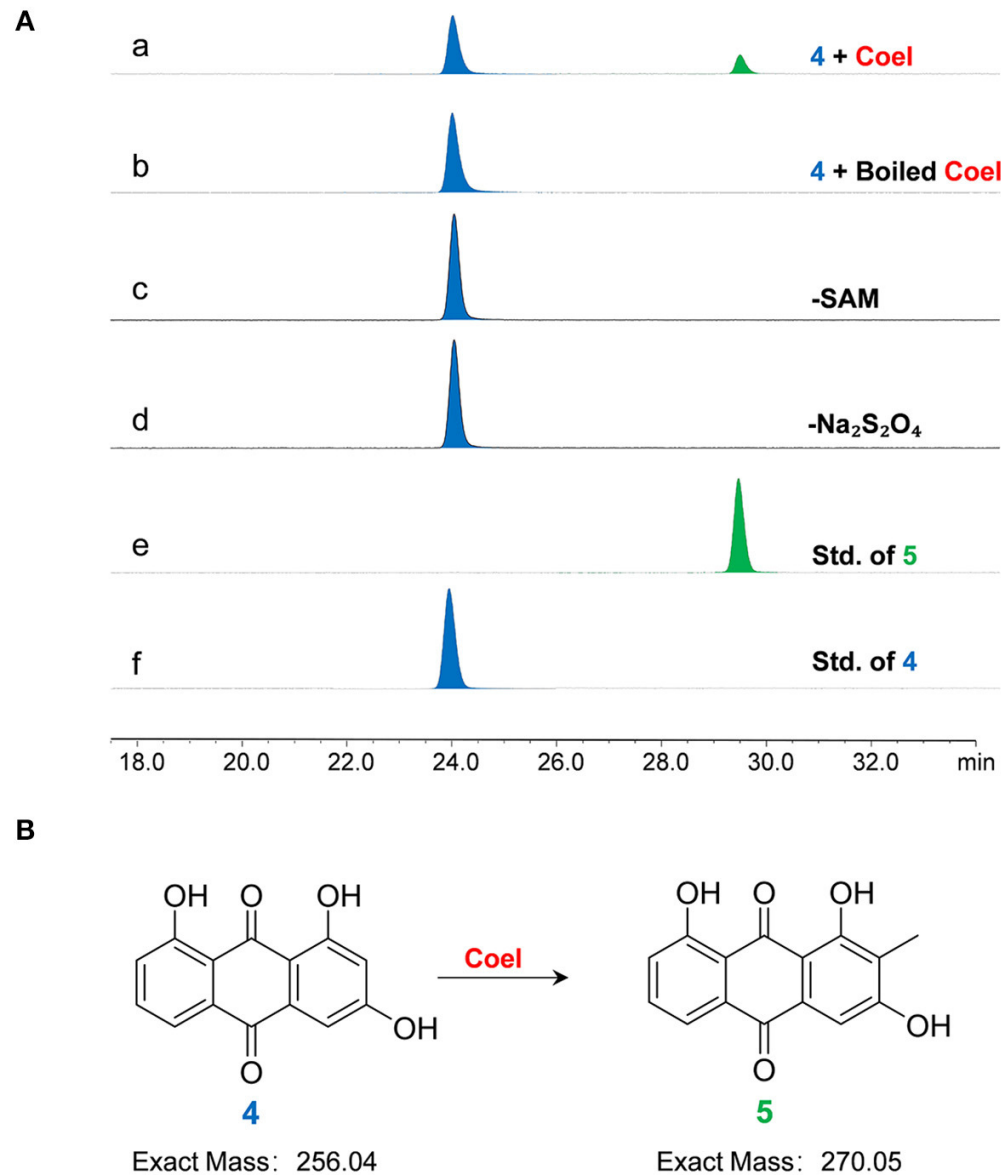
**(A) The** HPLC analysis of enzymatic assays of CoeI with substrate **4**. **(a)** The enzymatic assay of CoeI; **(b)** a reaction with boiled CoeI; **(c,d)** respective reactions without SAM and Na_2_S_2_O_4_; **(e)** the standard of product **5**; and **(f)** the standard of substrate **4**. Enzymatic assays illustrated that CoeI can catalyze the generation of product **5** from substrate **4** and indicated that the reaction was both SAM- and Na_2_S_2_O_4_-dependent. **(B)** CoeI catalyzed the methylation of compound **4**.

The character of CoeI was studied using *in vitro* enzymatic assays. When product **4** was used as a substrate, it can be methylated by CoeI to form product **5** (Figure 4). Without SAM and Na_2_S_2_O_4_, product **4** could no longer be converted to product **5** by CoeI, indicating that the reaction was both SAM- and Na_2_S_2_O_4_-dependent (Figure 4A).

However, unlike whole-cell transformation experiments, the generation of products **2** and **3** could not be detected in the enzymatic assay system, even after increasing the amount of enzyme and prolonging the reaction time. Therefore, product **5** was used as a substrate to test the activity of CoeI, but no new peaks were detected on HPLC (Supplementary Figure S2).

**Figure 5:**
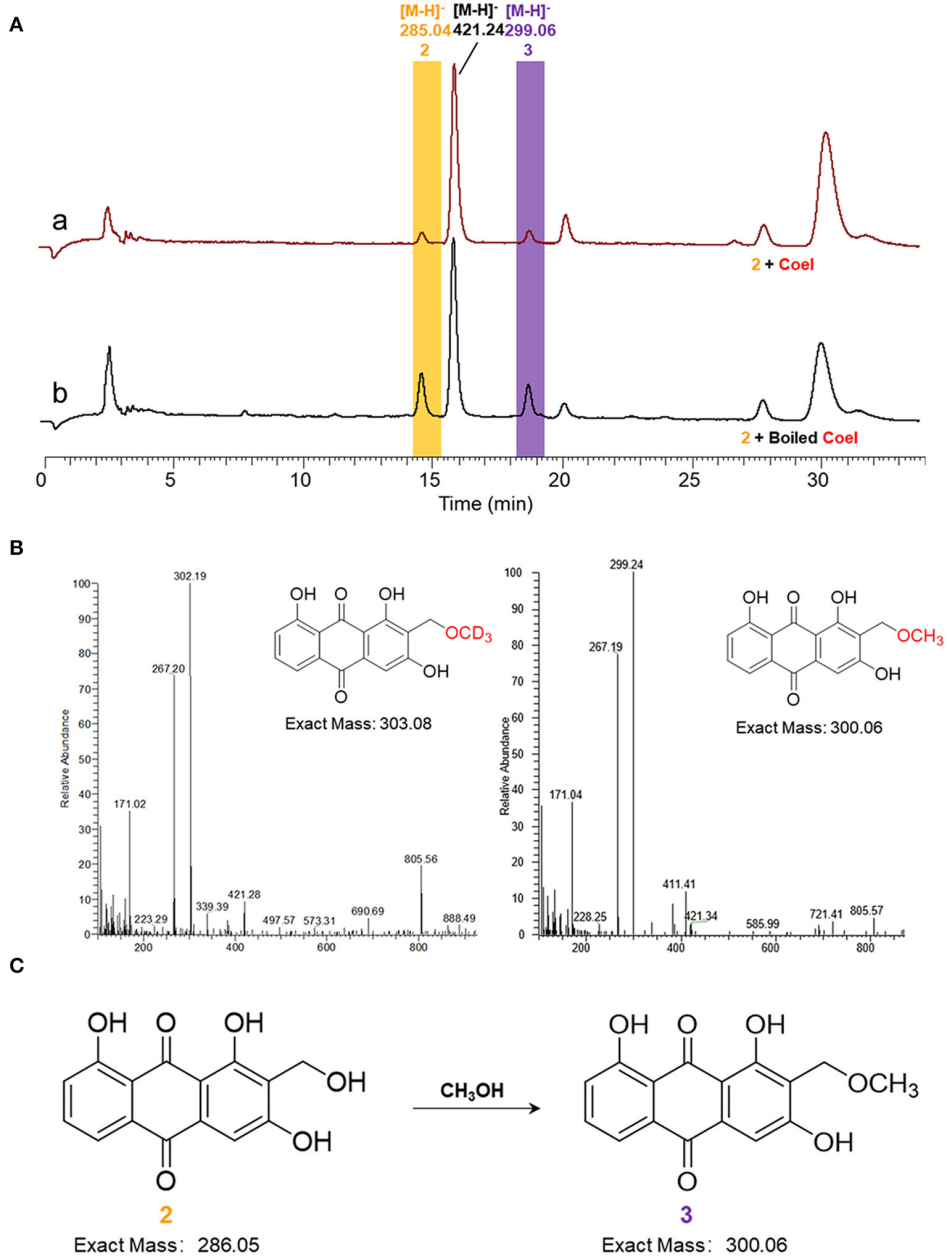
**(A)** HPLC analysis of enzymatic assays of CoeI to substrate **2**. **(a)** The enzymatic assay of CoeI to substrate **2** and **(b)** a reaction with boiled CoeI. **(B)** The origin of the methoxyl group in compound **3** was speculated by isotope labeling experiments. **(C)** Substrate **2** could transform into substrate **3** in methanol.

*In vitro* biochemical activity of CoeI toward substrate **2** was tested. Many peaks appeared in the HPLC profile of the living system. The formation of substrate **3** in the reaction system could be detected ([M–H]^−^ = 299.06), but the reaction could still proceed with boiled CoeI (Figure 5A), implying that substrates **2** and **3** occurred nonenzymatically. In addition, a new peak of [M–H]^−^ = 421.24 had a higher yield than that of substrate **3** (Figure 5A). To confirm the source of the methyl group of substrate **3**, we individually removed SAM and DTT from the system. The reaction could still proceed without adding SAM to the system (Supplementary Figures S4, S5), implying that the source of the methyl group of substrate **3** was not SAM. After removal of DTT from the reaction system, the peak of [M–H]^−^ = 421.24 disappeared. We speculated that the production of this compound was related to DTT (Supplementary Figure S3).

Given that the source of the methyl group of substrate **3** is not SAM, the source of the methyl group is probably methanol used to quench the reaction. To verify this conjecture, the reaction mixture was extracted with ethyl acetate instead of quenching with methanol. The production of substrate **3** could no longer be detected as a result of the reaction. In addition, perdeuterated methanol was used to quench the reaction. In accordance with expectations, substrate **3** was produced with an increased molecular weight of 3 (Figure 5B), confirming that the source of the methoxyl group in substrate **3** was methanol, which is used to quench the reaction (Figure 5C).

## Discussion

Bacteria are an important source of bioactive aromatic polyketides and many bacterial aromatic polyketides, and their derivatives have been used as drugs for the treatment of various acute and chronic diseases (Zhang et al., 2008; Zhan, 2009; Husain et al., 2020). Here, using a metagenomics approach, we cloned a type II polyketide BGC and obtained anthraquinone polyketide compounds by the heterologous expression of BGC in the *Streptomyces* host. Polyketides **2** and **3** have been isolated from plants before, and their modifications at C2 are significant (Bowie et al., 1962). Gene knockout and biochemical investigation revealed the radical SAM enzyme CoeI and a spontaneous methylation process.

Bioinformatic analysis revealed that CoeI belonged to the family of HemN-like enzymes and contained a highly conserved CxxxCxxC motif that coordinated the [4Fe−4S] cluster for binding and reductive cleavage of SAM (Sofia et al., 2001). Phylogenetic analysis of CoeI with other HemN-like enzymes revealed that, although they shared structural similarities, they could catalyze a variety of reactions to form important bioactive compounds (Jin et al., 2020). The knockout of the *coeI* gene prevented the biosynthesis of **2** and **3** and accumulated the intermediate **4**, which was a de-hydroxymethyl form of **2**, confirming that CoeI was involved in C2 modification during the biosynthesis of **2** and **3**.

When **4** was fed into the whole-cell transformation mixture, **2**, **3**, and **5**, the C2 methylated form of **4**, were identified (Supplementary Figure S3). However, unlike whole-cell transformation experiments, the generation of **2** and **3** could not be detected in the enzymatic assay system. Therefore, **5** was used as a substrate to test the activity of CoeI; however, no product was detected (Supplementary Figure S2). We referred to *in vitro* biochemical experiments of formation of cyclopropyl in CC-1065, the main product of which was also a methylated product of the substrate; however, this was not an authentic intermediate in the CC-1065 biosynthetic pathway but rather a by-product (Hiratsuka et al., 2014). It was suspected that **5** was not a true substrate for CoeI but rather a byproduct generated during the *in vitro* assay.

In contrast, the *in vitro* assay of CoeI enzyme using compound **2** as a substrate revealed that compound **2** was capable of converting to compound **3** in methanol without CoeI (Figure 5). Then, we speculated the mechanism by which compound **3** was generated. The C2 hydroxymethyl group of compound **2** was unstable and was prone to spontaneous dehydration with the formation of an α,β-unsaturated ketone, which was a reactive electrophile and could be attacked by CH_3_O^−^ dissociated from methyl to finally afford compound **3**. Na_2_S_2_O_4_ may act as a base to facilitate the dissociation of methanol to initiate the reaction during this process. Due to this mechanism, compound **2** could also react with other nucleophiles in the reaction mixture. Therefore, the new peaks found in the reaction mixture are products derived from the nucleophiles in the mixture (Figure 5A). DTT, as an active nucleophile in the reaction, should react with compound **2** to form **2**-DTT. As DTT is a more active nucleophile than methanol, the product of **2**-DTT obtained from the reaction was much higher than that of compound **3** (Figure 5A). We removed DTT from the reaction system, but eventually failed to detect the product of compound **2**, probably because compound **2** formed as a reaction with other nucleophilic species in the system.

**Figure 6:**
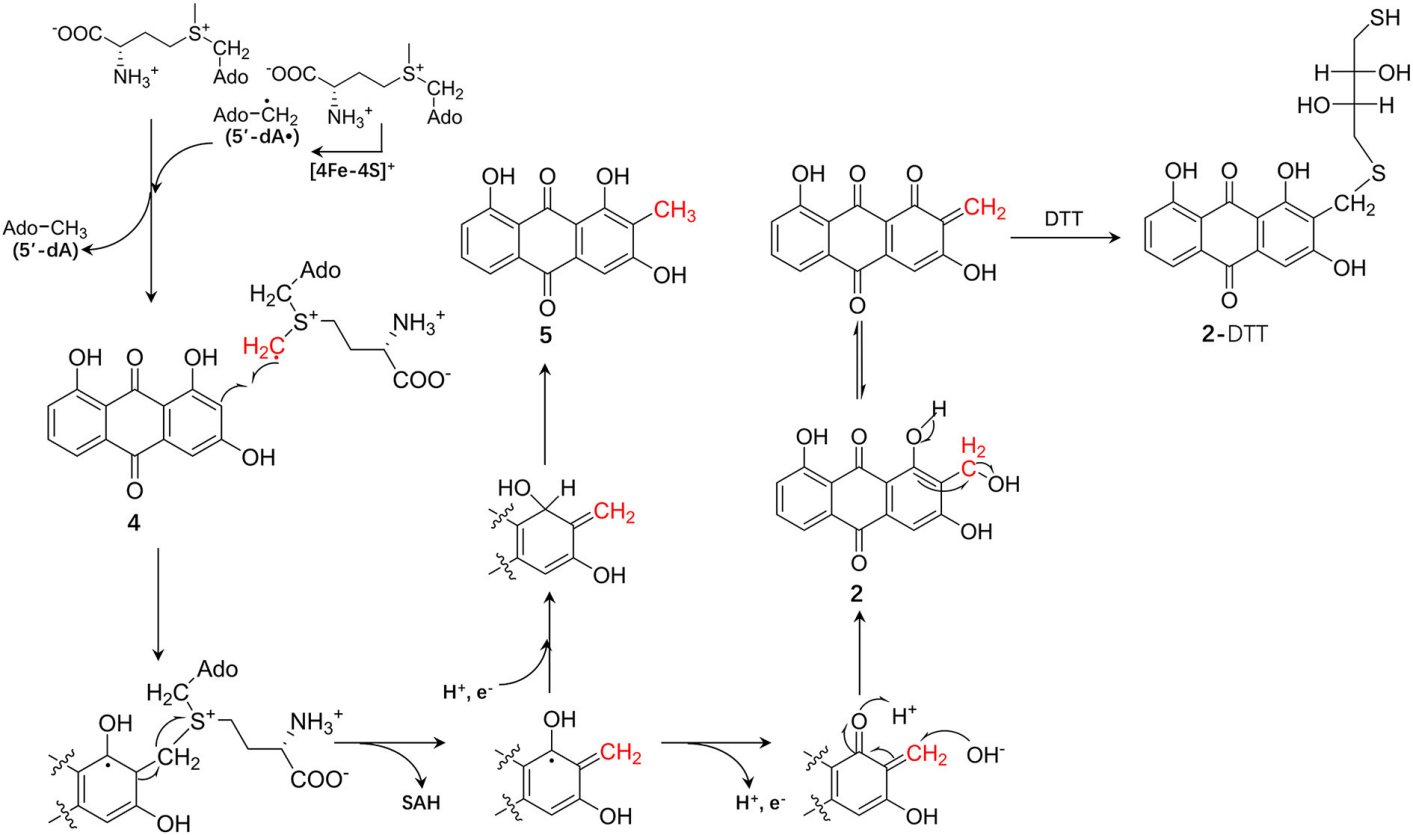
The proposed chemical mechanism of the CoeI-catalyzed transformation of **4**–**5** and **2**, and the formation mechanism of **2**-dithiothreitol (DTT).

Based on these results, we speculated on the hydroxymethylation process of compound **4** catalyzed by CoeI: first, two molecules of SAM were consumed to generate the SAM methylene radical. Second, the adduct of compound **4** and SAM was obtained as a result of the addition reaction between the SAM methylene radical and compound **4**, and then, the methylene radical of **4** was obtained by an electronic rearrangement to release one molecule of SAH. In the final process, if the methylene radical of compound **4** acquired one proton and one electron, compound **5** was formed as a result of an electronic rearrangement. If the methylene radical of compound **4** lost one proton and one electron, an α,β-unsaturated ketone intermediate would be formed, and the intermediate would be attacked by a hydroxyl group to form a hydroxymethylated product **2**. As the hydroxymethyl group in product **2** was structurally unstable and could easily be dehydrated to form an α,β-unsaturated ketone intermediate, this intermediate might be attacked by the methoxy anion dissociated by DTT to form **2**-DTT (Figure 6). As the hydroxymethylated product **2** was not directly detected in the *in vitro* reaction system, further optimization of the reaction system or substrate–protein cocrystallization would demonstrate the mechanism in detail. In the study of the catalytic function of the enzyme NosN in the biosynthesis of nosiheptide, a similar catalytic activity of hydroxymethylation on *sp*^2^ carbon was also observed, but this hydroxymethylated product was considered as a shunt product in the catalytic process (LaMattina et al., 2017). Moreover, the Booker Lab provided evidence for the partitioning of different reaction outcomes (methylation vs. lactone formation) based on the reductant used in the reaction (Wang et al., 2019).

In summary, a type II polyketide COE BGC was identified from the soil metagenome. Coelulatins **2** and **3**, which were previously reported as plant metabolites, were obtained by the heterologous expression of COE BGC in the *S. coelicolor* M1146 host. Most interestingly, a radical SAM enzyme in this PSK-II, CoeI, with a methylation function was characterized. This may also play a key role in the hydroxymethylation of anthraquinone. It is also worth noting that we did not know whether the formation of coelulatin **2** was directly completed by the enzyme CoeI. To solve this problem, it is necessary to analyze the crystal structure of CoeI and its subsequent complexes. This study enriched the investigation of the biosynthetic pathways of aromatic polyketides and the catalytic types of the HemN-like radical SAM methyltransferase family.

## Data availability statement

The data presented in the study are deposited in the GenBank repository, accession number MN601984–MN601997.

## Author contributions

ZF designed the experiments. LN and TW performed the experiments. LN and MC performed NMR measurements. LN, TW, YL, SW, and ZF analyzed the experimental results. LN and ZF wrote the manuscript. All authors have read and agreed to the final version of the manuscript.

## Funding

This study was supported by the National Natural Science Foundation of China (Grant No. 31770049) and the State Key Laboratory of Bio-Organic and Natural Products Chemistry (SKLBNPC20349).

## Conflict of interest

The authors declare that the research was conducted in the absence of any commercial or financial relationships that could be construed as a potential conflict of interest.

## Publisher's note

All claims expressed in this article are solely those of the authors and do not necessarily represent those of their affiliated organizations, or those of the publisher, the editors and the reviewers. Any product that may be evaluated in this article, or claim that may be made by its manufacturer, is not guaranteed or endorsed by the publisher.

## References

[B1] Bauerle M. R. Schwalm E. L. Booker. S. J. (2015). Mechanistic diversity of radical S-adenosylmethionine (SAM)-dependent methylation. J. Biol. Chem. 290, 3995–4002. 10.1074/jbc.R114.607044 25477520 PMC4326810

[B2] Bierman M. Logan R. O'Brien K. Seno E. T. Rao R. N Schoner B. E. (1992). Plasmid cloning vectors for the conjugal transfer of DNA from *Escherichia coli* to *Streptomyces spp*. Gene 116, 43–49. 10.1016/0378-1119(92)90627-2 1628843

[B3] Bowie J. H. Cooke R. G. Wilkin P. E. (1962). Colouring matters of Australian plants. *X. Anthraquinones* from Coelospermum species. Aust. J. Chem. 15, 337–341. 10.1071/CH9620336

[B4] Broderick J. B. Duffus B. R. Duschene K. S. Shepard E. M. (2014). Radical S-adenosylmethionine enzymes. *Chem*. Rev. 114, 4229–4317. 10.1021/cr4004709 24476342 PMC4002137

[B5] Broderick W. E. Broderick J. B. (2019). Radical SAM enzymes: surprises along the path to understanding mechanism. J. Biol. Inorg. Chem. 24, 769–776. 10.1007/s00775-019-01706-w 31494759 PMC8837180

[B6] Buckel W. Thauer R. K. (2011). Dual role of S-adenosylmethionine (SAM+) in the methylation of *sp*^2^-hybridized electrophilic carbons. Angew. Chem. Int. Ed. Engl. 50, 10492–10494. 10.1002/anie.201105076 21919174

[B7] Cheng J. D. Liu W. Q. Zhu X. Y. Zhang Q. (2022). Functional diversity of HemN-like proteins. ACS Bio. Med. Chem. Au. 2, 109–119. 10.1021/acsbiomedchemau.1c00058 PMC10114718 37101745

[B8] Feng Z. Y. Kallifidas D. Brady S. F. (2011). Functional analysis of environmental DNA-derived type II polyketide synthases reveals structurally diverse secondary metabolites. *Proc. Natl Acad*. Sci U. S. A. 108, 12629–12634. 10.1073/pnas.1103921108 21768346 PMC3150919

[B9] Frey P. A. Hegeman A. D. Ruzicka F. J. (2008). The radical SAM superfamily. Crit. Rev. Biochem. Mol. Biol. 43, 63–88. 10.1080/10409230701829169 18307109

[B10] Fujimori D. G. (2013). Radical SAM-mediated methylation reactions. Curr. Opin. Chem. Biol. 17, 597–604. 10.1016/j.cbpa.2013.05.032 23835516 PMC3799849

[B11] Gust B. Kieser T. Chater K. (2002). REDIRECT© technology: PCR-targeting system in Streptomyces coelicolor. John Innes Centre 3, 1–42.

[B12] Hiratsuka T. Suzuki H. Kariya R. Seo T. Minami A. Oikawa H.. (2014). Biosynthesis of the structurally unique polycyclopropanated polyketide-nucleoside hybrid jawsamycin (FR-900848). Angew. Chem. Int. Ed. Engl. 53, 5423–5429. 10.1002/anie.201402623 24756819

[B13] Holliday G. L. Akiva E. Meng. E. C Brown S. D. Calhoun S. Pieper U.. (2018). Atlas of the radical SAM superfamily: divergent evolution of function using a “plug and play” domain. Methods Enzymol. 606, 1–71. 10.1016/bs.mie.2018.06.004 30097089 PMC6445391

[B14] Huang W. Xu H. Li Y. Zhang F. Chen X. Y. He Q. L.. (2012). Characterization of yatakemycin gene cluster revealing a radical S-adenosylmethionine dependent methyltransferase and highlighting spirocyclopropane biosynthesis. J. Am. Chem. Soc. 134, 8831–8840. 10.1021/ja211098r 22612591

[B15] Husain S. M. Prag A. Linnenbrink A. Bechthold A. Muller M. (2020). Insights into the role of ketoreductases in the biosynthesis of partially reduced bacterial aromatic polyketides. Chem. Bio. Chem. 21, 780–784. 10.1002/cbic.201900357 31507033 PMC7154522

[B16] Jansson A. Niemi J. Lindqvist Y. Mäntsäl,ä P. Schneider G. (2003). Crystal structure of aclacinomycin-10-hydroxylase, a S-adenosyl-L-methionine-dependent methyltransferase homolog involved in anthracycline biosynthesis in *Streptomyces purpurascens*. J. Mol. Biol. 334, 269–280. 10.1016/j.jmb.2003.09.061 14607118

[B17] Javidpour P. Bruegger J. Srithahan S. Korman T. P. Crump M. P. Crosby J.. (2013). The determinants of activity and specificity in actinorhodin type II polyketide ketoreductase. Chem. Biol. 20, 1225–1234. 10.1016/j.chembiol.2013.07.016 24035284 PMC3855848

[B18] Jin W. B. Wu S. Jian X. H. Yuan H. Tang G. L. (2018). A radical S-adenosyl-L-methionine enzyme and a methyltransferase catalyze cyclopropane formation in natural product biosynthesis. Nat. Commun. 9, 2771–2781. 10.1038/s41467-018-05217-1 30018376 PMC6050322

[B19] Jin W. B. Wu S. Xu Y. F. Yuan H. Tang G. L. (2020). Recent advances in HemN-like radical S-adenosyl-l-methionine enzyme-catalyzed reactions. Nat. Prod. Rep. 37, 17–28. 10.1039/C9NP00032A 31290896

[B20] Krupa J. Lessmann H. Lackner H. (1989). Ein a-Methylanthrachinon aus *Streptomyceten*. Liebigs Ann. Chem. 1989, 699–701. 10.1002/jlac.198919890217

[B21] LaMattina J. W. Nix D. B. Lanzilotta W. N. (2016). Radical new paradigm for heme degradation in *Escherichia coli* O157:H7. Proc. Natl Acad. Sci. U. S. A. 113, 12138–12143. 10.1073/pnas.1603209113 27791000 PMC5087033

[B22] LaMattina J. W. Wang B. Badding E. D. Gadsby L. K. Grove T. L. Booker S. J. (2017). NosN, a radical S-Adenosylmethionine methylase, catalyzes both C1 transfer and formation of the ester linkage of the side-ring system during the biosynthesis of Nosiheptide. J. Am. Chem. Soc. 139, 17438–17445. 10.1021/jacs.7b08492 29039940 PMC5938625

[B23] Layer G. Moser J. Heinz D. W. Jahn D. Schubert W. D. (2003). Crystal structure of coproporphyrinogen III oxidase reveals cofactor geometry of Radical SAM enzymes. EMBO J. 22, 6214–6224. 10.1093/emboj/cdg598 14633981 PMC291839

[B24] Layer G. Verfurth K. Mahlitz E. Jahn D. (2002). Oxygen-independent coproporphyrinogen-III oxidase HemN from *Escherichia coli*. J. Biol. Chem. 277, 34136–34142. 10.1074/jbc.M205247200 12114526

[B25] Mahanta N. Zhang Z. Hudson G. A. van der Donk W. A. Mitchell D. A. (2017). Reconstitution and substrate specificity of the radical S-Adenosyl-methionine thiazole C-Methyltransferase in thiomuracin biosynthesis. J. Am. Chem. Soc. 139, 4310–4313. 10.1021/jacs.7b00693 28301141 PMC5477235

[B26] Mehta A. P. Abdelwahed S. H. Mahanta N. Fedoseyenko D. Philmus B. Cooper L. E.. (2015). Radical S-adenosylmethionine (SAM) enzymes in cofactor biosynthesis: a treasure trove of complex organic radical rearrangement reactions. J. Biol. Chem. 290, 3980–3986. 10.1074/jbc.R114.623793 25477515 PMC4326808

[B27] Musiol E. M. Hartner T. Kulik A. Moldenhauer J. Piel J. Wohlleben W.. (2011). Supramolecular templating in kirromycin biosynthesis: the acyltransferase KirCII loads ethylmalonyl-CoA extender onto a specific ACP of the trans-AT PKS. Chem. Biol. 18, 438–444. 10.1016/j.chembiol.2011.02.007 21513880

[B28] Ruszczycky M. W. Zhong A. Liu H. W. (2018). Following the electrons: peculiarities in the catalytic cycles of radical SAM enzymes. Nat. Prod. Rep. 35, 615–621. 10.1039/C7NP00058H 29485151 PMC6051909

[B29] Ryan W. K. John D. B. Brady S. F. (2009). An environmental DNA-derived type II polyketide biosynthetic pathway encodes the biosynthesis of the novel pentacyclic polyketide, erdacin. Angew. Chem. Int. Ed. Engl. 48, 6257–6261. 10.1002/anie.200901209 19621341 PMC2930765

[B30] Sinner E. K. Marous D. R. Townsend C. A. (2022). Evolution of methods for the study of Cobalamin-dependent radical SAM enzymes. ACS Bio. Med. Chem. Au. 2, 4–10. 10.1021/acsbiomedchemau.1c00032 35341020 PMC8950095

[B31] Sofia H. J. Chen G. Hetzler B. G. Reyes-Spindola J. F. Miller N. E. (2001). Radical SAM, a novel protein superfamily linking unresolved steps in familiar biosynthetic pathways with radical mechanisms: functional characterization using new analysis and information visualization methods. Nucleic Acids Res. 29, 1097–1106. 10.1093/nar/29.5.1097 11222759 PMC29726

[B32] Wang B. LaMattina J. W. Marshall S. L. Booker S. J. (2019). Capturing intermediates in the reaction catalyzed by NosN, a class C radical S-Adenosylmethionine methylase involved in the biosynthesis of the Nosiheptide side-ring system. J. Am. Chem. Soc. 141, 5788–5797. 10.1021/jacs.8b13157 30865439 PMC7061316

[B33] Wang J. Zhang R. Chen H. Sun X. Yan X. X. Shen Y. J.. (2020). Biosynthesis of aromatic polyketides in microorganisms using type II polyketide synthases. Microb. Cell Fact. 19, 110–122. 10.1186/s12934-020-01367-4 32448179 PMC7247197

[B34] Wang S. Gao X. Gao Y. J. Li Y. D. Cao M. M. Xi Z. H.. (2017). Tetracycline resistance genes identified from distinct soil environments in China by functional metagenomics. Front. Microbiol. 8, 1406–1415. 10.3389/fmicb.2017.01406 28790997 PMC5522880

[B35] Wawrik B. Kerkhof L. Zylstra G. J. Kukor J. J. (2005). Identification of unique type II polyketide synthase genes in soil. Appl. Environ. Microbiol. 71, 2232–2238. 10.1128/AEM.71.5.2232-2238.2005 15870305 PMC1087561

[B36] Wu S. Jian X. Yuan H. Jin H. Yin W. B. Wang Y. J.. (2017). Unified biosynthetic origin of the benzodipyrrole subunits in CC-1065. ACS Chem. Biol. 12, 1603–1610. 10.1021/acschembio.7b00302 28426198

[B37] Zhan J. X. (2009). Biosynthesis of bacterial aromatic polyketides. Curr. Top. Med. Chem. 9, 1598–1610. 10.2174/156802609789941906 19903160

[B38] Zhang Q. Van Der Donk W. A. Liu W. (2012). Radical-mediated enzymatic methylation: a tale of two SAMs. Accounts Chem. Res. 45, 555–564. 10.1021/ar200202c 22097883 PMC3328197

[B39] Zhang W. J. Li Y. R. Tang Y. (2008). Engineered biosynthesis of bacterial aromatic polyketides in Escherichia coli. PNAS 105, 20683–20688. 10.1073/pnas.0809084105 19075227 PMC2634872

