# Supplementary material for: Biosynthesis of coelulatin for the methylation of anthraquinone featuring HemN-like radical S-adenosyl-L-methionine enzyme
Source: Front Microbiol. 2022 Nov 17;13:1040900. doi: 10.3389/fmicb.2022.1040900 (PMC9714029; doi:10.3389/fmicb.2022.1040900)
Supplement: Supplementary file 1 [file Data_Sheet_1.pdf]

Biosynthesis of coelulatin for the methylation of anthraquinone featuring HemN-like radical S-adenosyl-L-methionine enzyme

Lishuang Nie[1], Tianyi Wei[2], Mingming Cao[1], Yunbin Lyu[1], Shaochen Wang[1], and Zhiyang Feng[1]*

1 College of Food Science and Technology, Nanjing Agricultural University, 1Weigang, Nanjing 210095, China;
2 State Key Laboratory of Bio-organic and Natural Products Chemistry, Shanghai Institute of Organic Chemistry, Chinese Academy of Sciences, 345 Lingling Road, Shanghai 200032, China
* Correspondence: zfeng@njau.edu.cn

Table S1. Primers used in this study S1
Table S2. ^{1}H (600 MHz) and ^{13}C (150 MHz) NMR data of compounds **1**, **2** and **3** in DMSO-d_6 .... S2
Table S3. ^{1}H (600 MHz) and ^{13}C (150 MHz) NMR data of compounds **4** and **5** in DMSO-d_6 .... S3
Figure S1. Sequence alignments for CoeI with homologous proteins. S4
Figure S2. Enzymatic assays of CoeI to substrate **5** under aerobic conditions S5
Figure S3. **(A)** Whole cell transformation experiments using substrates **4** and **5**; **(B)** **4** can generate **5** and **2** through whole cell transformation experiments, but **5** cannot generate **2** through whole cell transformation experiments S6
Figure S4. HPLC analysis of enzymatic assays of CoeI to substrate **2** S7
Figure S5. MS spectrum of compound **2**-DTT S8
Figure S6. Detection of the production of **2**-DTT in the enzyme activity assay of **4** S9
Figure S7. MS spectrum of compound **1** S10
Figure S8. MS spectrum of compound **2** S11
Figure S9. MS spectrum of compound **3** S12
Figure S10. MS spectrum of compound **4** S13
Figure S11. MS spectrum of compound **5** S14
Figure S12. ^{1}H NMR of compound **1** S15
Figure S13. ^{13}C NMR of compound **1** S15
Figure S14. ^{1}H-^{1}H NOESY data of compound **1** S16
Figure S15. HMQC data of compound **1** S16

Figure S16. HMBC data of compound **1** S17
Figure S17. ^{1}H NMR of compound **2**.......... S17
Figure S18. ^{13}C NMR of compound **2** S18
Figure S19. ^{1}H-^{1}H NOESY data of compound **2** S18
Figure S20. HMQC data of compound **2** S19
Figure S21. HMBC data of compound **2** S19
Figure S22. ^{1}H NMR of compound **3**.......... S20
Figure S23. ^{13}C NMR of compound **3** S20
Figure S24. ^{1}H NMR of compound **4**.......... S21
Figure S25. ^{13}C NMR of compound **4** S21
Figure S26. ^{1}H-^{1}H NOESY data of compound **4** S22
Figure S27. HMQC data of compound **4** S22
Figure S28. HMBC data of compound **4** S23
Figure S29. ^{1}H NMR of compound **5**.......... S23
Figure S30. ^{13}C NMR of compound **5** S24
Figure S31. ^{1}H-^{1}H NOESY data of compound **5** S24
Figure S32. HMQC data of compound **5** S25
Figure S33. HMBC data of compound **5** S25

Table S1. Primers used in this study

Primers	Sequence (5'→ 3')
540-F	GGNTGCACSTCNGGNMTSGAC
1100-R	CCGATSGCNCCSAGNGAGTG
coeB-F	ATGACAGCCGGAATGGCCACCGGTCCCGGGGGCCGCACCACTAG TGGCGCGCCGGAATA
coeB-R	TCACTGAGGCGCGGCCTCGCGGACCACGATGGCGGAGTTACTAG TTTAATTAATGCAG
coeI-F	ATGACAGCGCTGGAGATCGCCATGGCCGGCGACCCGTATACTAGT GGCGCGCCGGAATA
coeI-R	TCAGCCATGGTCGGCCACCTTCTCTGCCTCGTTGGCCTCACTAGT TTAATTAATGCAG
orf10-F	ATGGCCTCCTGGAACCCTGGCCGGATGTTCGTCTCACTGACTAGT GGCGCGCCGGAATA
orf10-R	TCAGGCTGCATATCTGTGCTCGTTGATGACACCGCCGAGACTAGT TTAATTAATGCAG
orf11-F	ATGACGGGCCAACAAGGCACGGAGGCCGGCGTGCTCACCACTAG TGGCGCGCCGGAATA
orf11-R	TCAGCCTCCGACTCGGAGGTAGTGCCCCCAATACCCCTGACTAGT TTAATTAATGCAG
coeI protein-F	GGAATTCCATATGACAGCGCTGGAGATCGCCAT
coeI protein-R	CCGCTCGAG GCCATGGTCGGCCACCCTCT

Table S2. ^{1}H (600 MHz) and ^{13}C (150 MHz) NMR data of compounds **1**, **2** and **3** in DMSO-d_6

NO.	1		2		3	
	δ_C, Type	δ_H	δ_C, Type	δ_H	δ_C, Type	δ_H
1	142.3, C		164.5, C		165.7, C	
2	122.4, C		120.9, C		119.7, C	
3	161.2, C		163.4, C		164.1, C	
4	112.9, CH	7.54, s	108.8, CH	7.22, s	108.8, CH	7.24, s
4a	136.4, C		109.1, C		109.0, C	
5	118.6, CH	7.61, s	119.7, CH	7.65, s	117.3, CH	7.65, s
6	136.7, CH	7.70, s	137.2, CH	7.75, s	137.15, CH	7.75, s
7	124.8, CH	7.32, s	124.9, CH	7.33, s	124.95, CH	7.33, s
8	161.9, C		161.7, C		161.7, CH	
8a	117.3, C		116.1, C		116.2, CH	
9	189.7, C		190.7, C		190.4, CH	
9a	130.7, C		133.9, C		134.5, CH	
10	182.5, C		181.6, C		181.8, CH	
10a	132.9, C		133.5, C		133.6, CH	
1-CH_3	20.43, CH_3	2.51, s				
2-COOH	168.8, C					
2-CH_2OH			51.6, CH2	4.50, s		
2-CH_2OCH_3					58.1, CH_2	4.43, s
2-CH_2OCH_3					61.6, CH_3	2.61, s

Table S3. ^{1}H (600 MHz) and ^{13}C (150 MHz) NMR data of compounds **4** and **5** in DMSO-d_6

NO.	**4**		**5**	
	δ_C, **Type**	δ_H	δ_C, **Type**	δ_H
1	165.2, C		162.8, C	
2	108.4, CH	6.51, s	107.6, C	
3	167.6, C		163.6, C	
4	110.2, CH	7.08, s	113.0, CH	7.45, s
4a	108.8, C		109.1, C	
5	119.6, CH	7.63, s	121.8, CH	7.21, s
6	136.9, CH	7.73, s	135.7, CH	7.22, s
7	124.8, CH	7.32, s	125.2, CH	8.06, s
8	161.6, C		162.6, C	
8a	116.1, C		117.9, C	
9	189.8, C		186.2, C	
9a	135.4, C		132.3, C	
10	181.5, C		182.5, C	
10a	133.6, C		129.9, C	
2-CH_3			8.58, C	2.07, s

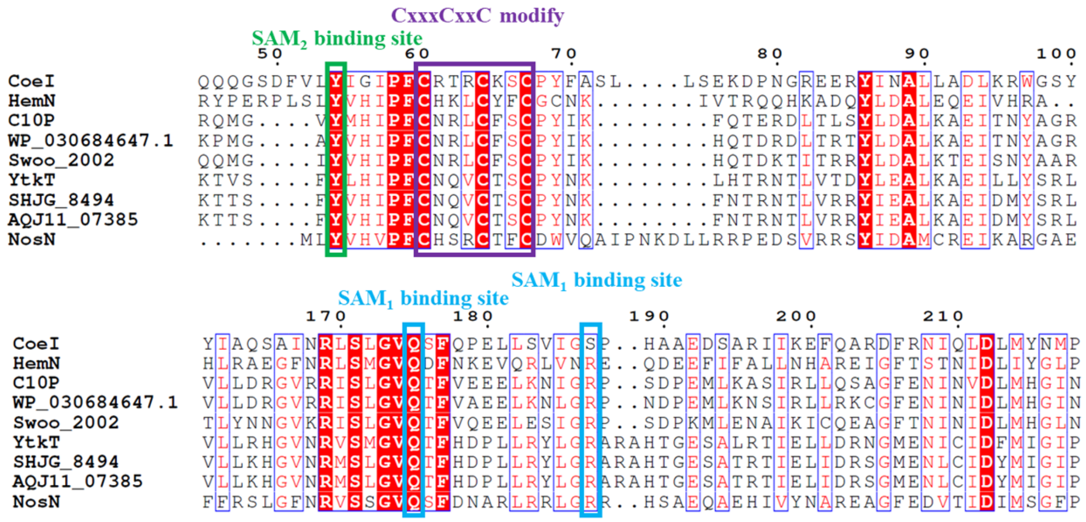

Figure S1 Sequence alignments for CoeI with homologous proteins. The alignment was carried out using ClustalW. The conserved motif CxxxCxxC for binding [4Fe-4S] cluster is marked in purple, the conserved glutamine and arginine residues for binding SAM_1 selected for mutation are marked in blue, and the conserved tyrosine residue for binding SAM_2 selected for mutation is marked in green.

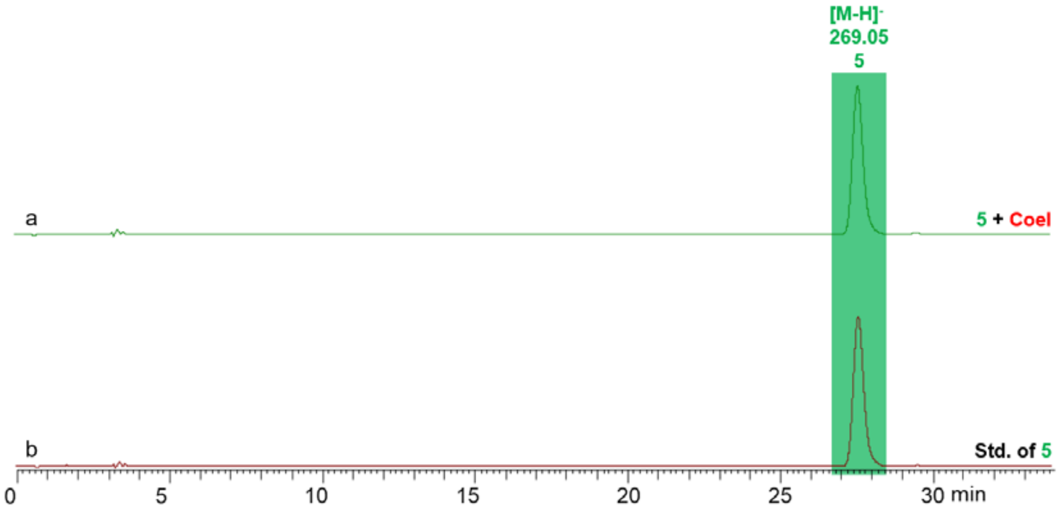

Figure S2. Enzymatic assays of CoeI to substrate **5** under aerobic conditions. a: HPLC analysis of enzymatic products of CoeI to substrate **5**. b: standard of **5**. No new peak was detected on HPLC.

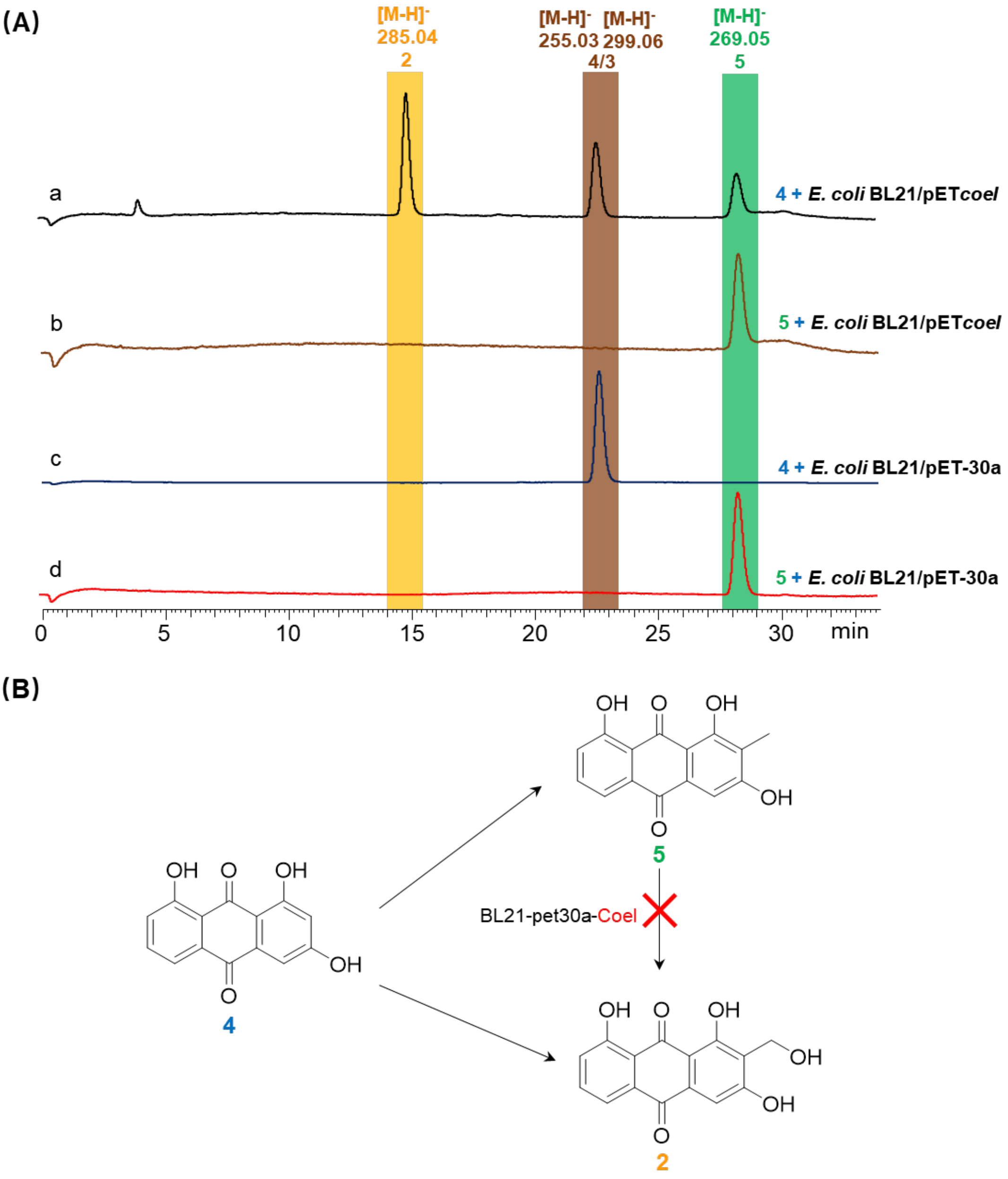

Figure S3. (A) Whole cell transformation experiments using substrates **4** and **5**. a: HPLC analysis of whole cell transformation experiment using substrate **4**. b: HPLC analysis of whole cell transformation experiment using substrate **5**. c: the control reaction using substrate **4** with *E. coli* BL21/pET-30a cells. d: the control reaction using substrate **5** with *E. coli* BL21/pET-30a cells. Whole cell transformation experiments revealed that substrate **4** could be converted to **2**, **3**, and a new compound **5** in the reaction system. **(B) 4** can generate **5** and **2** through whole cell transformation experiments, but **5** cannot generate **2** through whole cell transformation experiments.

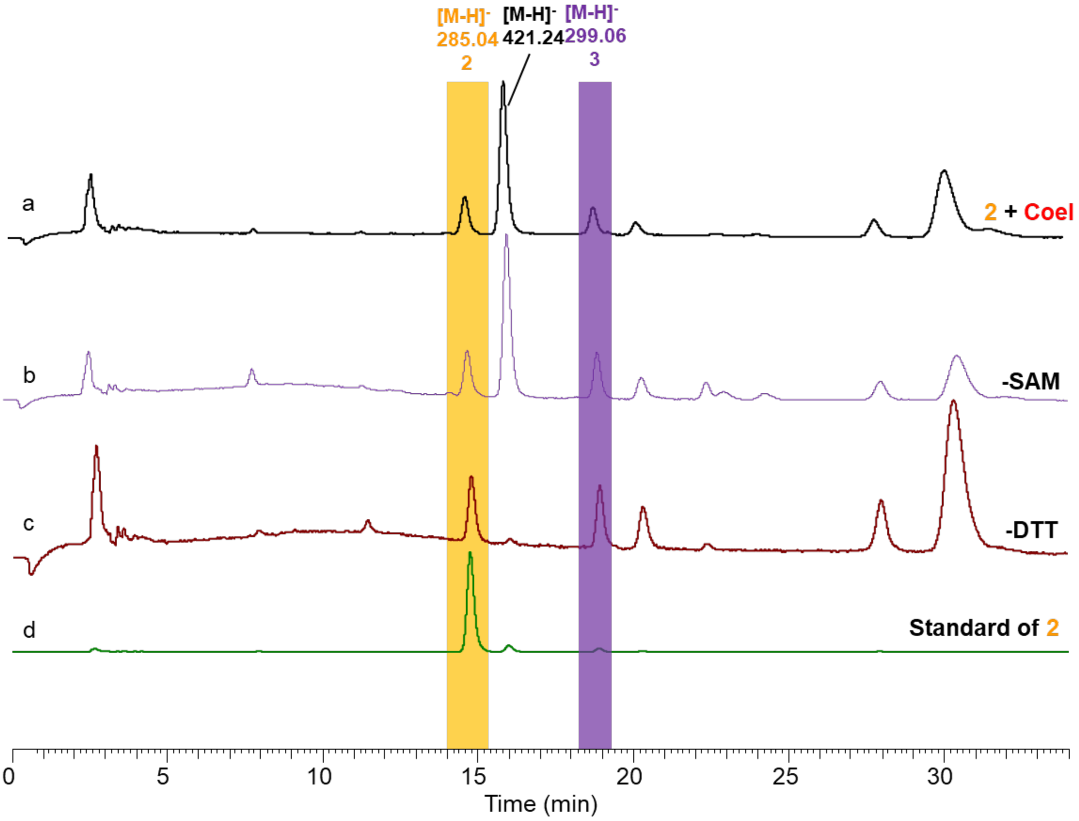

Figure S4. HPLC analysis of enzymatic assays of CoeI to substrate **2**. a: enzymatic assay of CoeI to substrate **2**. b: reaction without SAM. c: reaction without DTT. d: standard of **2**.

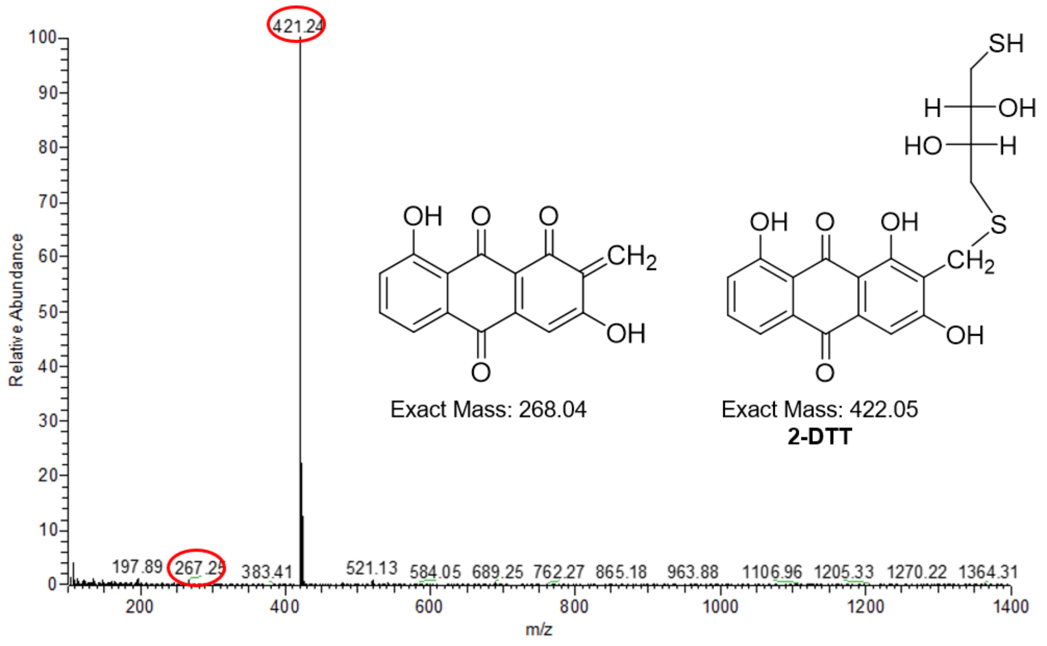

Figure S5. MS spectrum of compound **2-**DTT.

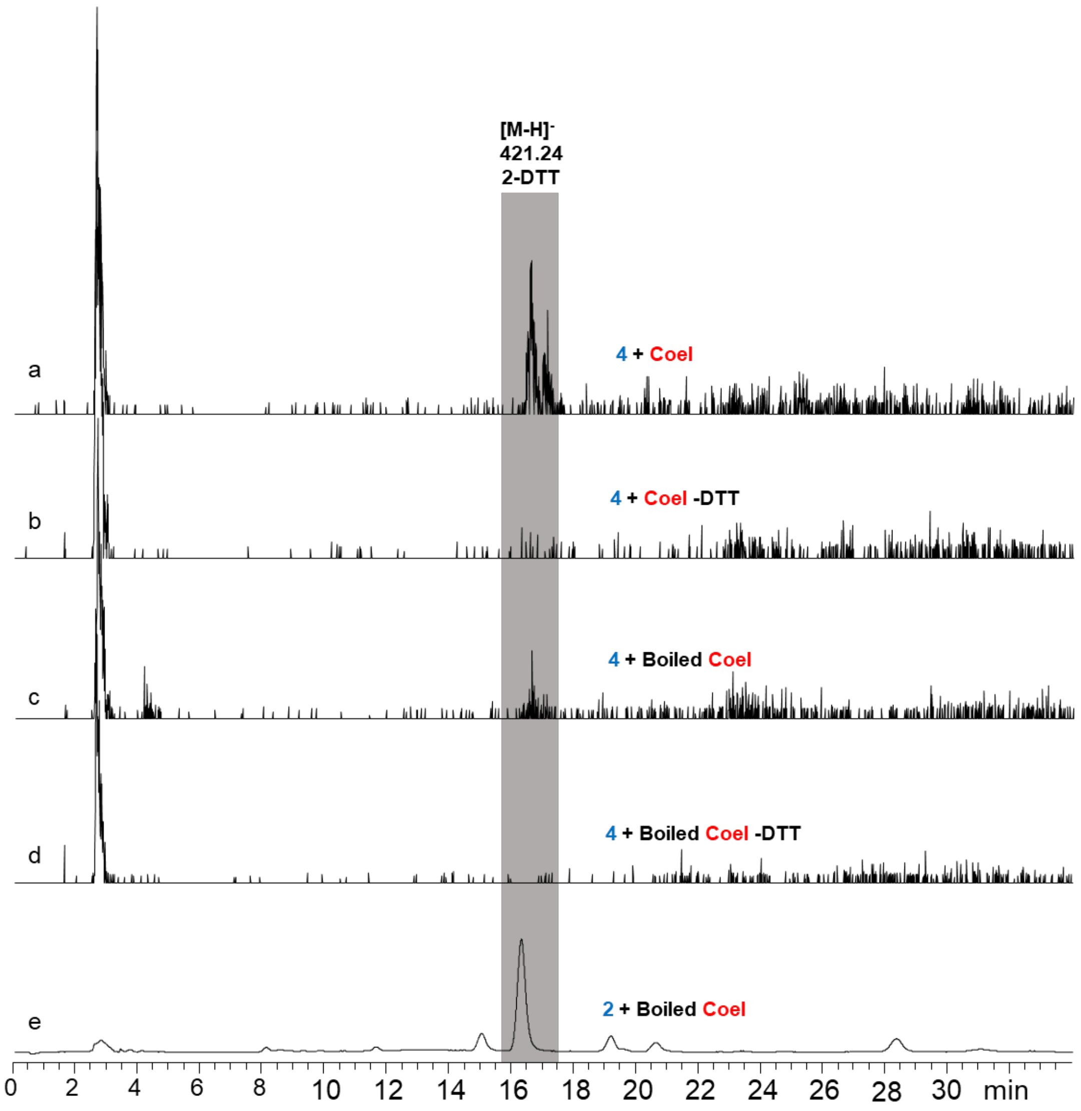

Figure S6. Detection of the production of **2**-DTT in the enzyme activity assay of **4**. a: LC-MS analysis of enzymatic products using substrate **4**. b: the control reaction without DTT. c: the control reaction with boiled CoeI. d: the control reaction with boiled CoeI and without DTT. e: HPLC analysis of enzymatic products using substrate **4** with boiled CoeI.

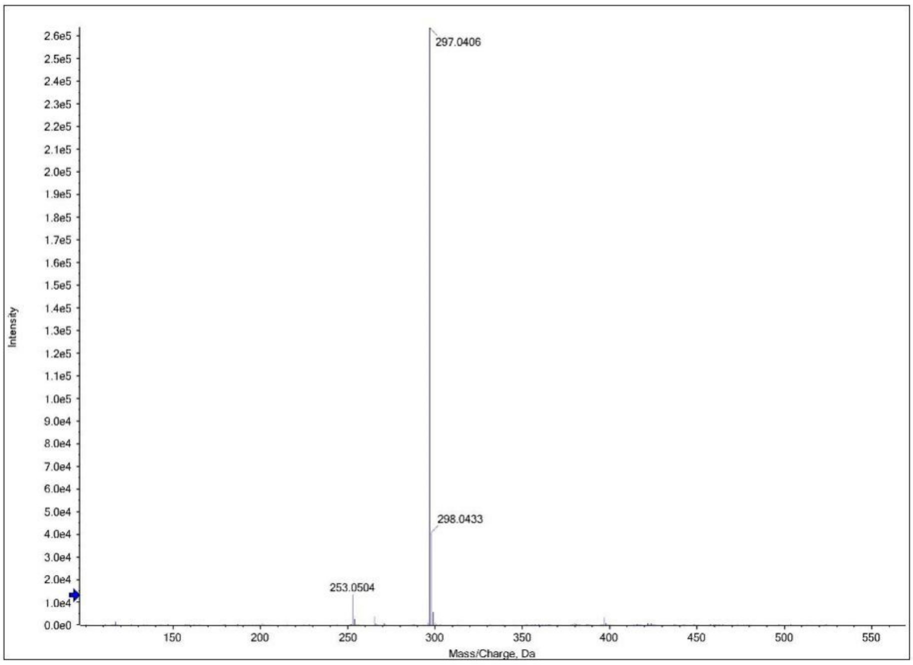

Figure S7. MS spectrum of compound **1**.

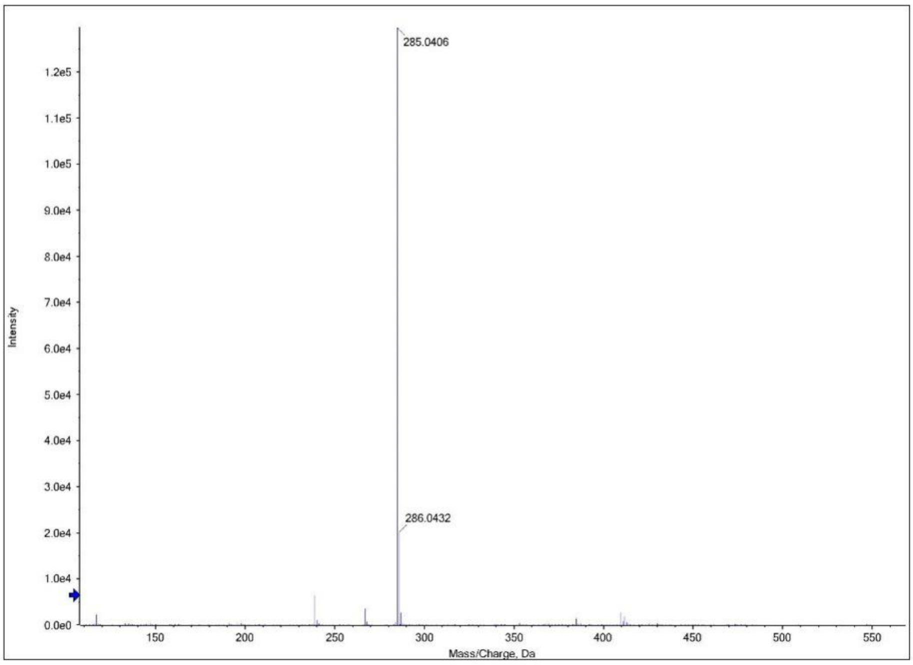

Figure S8. MS spectrum of compound **2**.

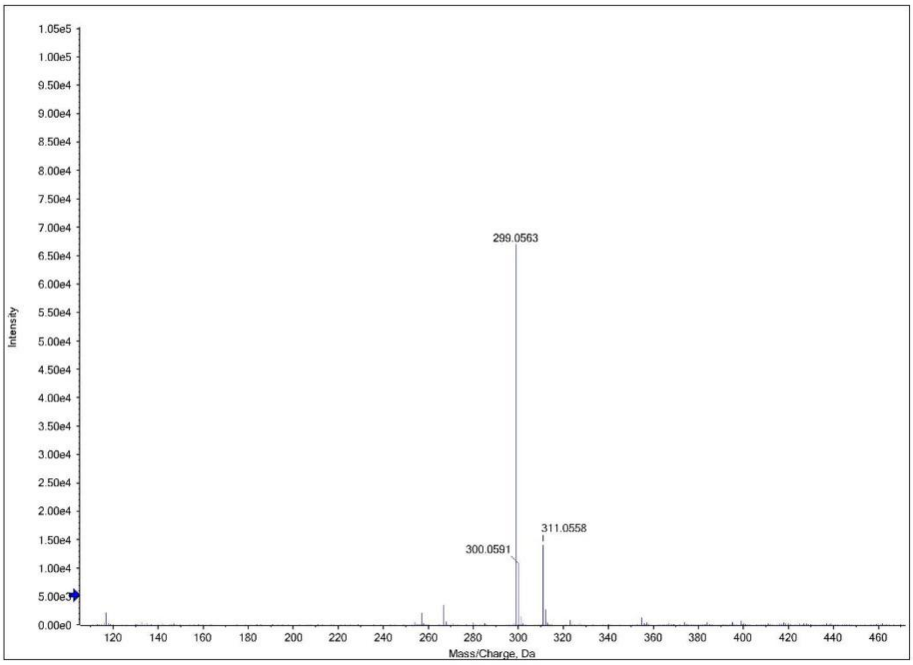

Figure S9. MS spectrum of compound **3**.

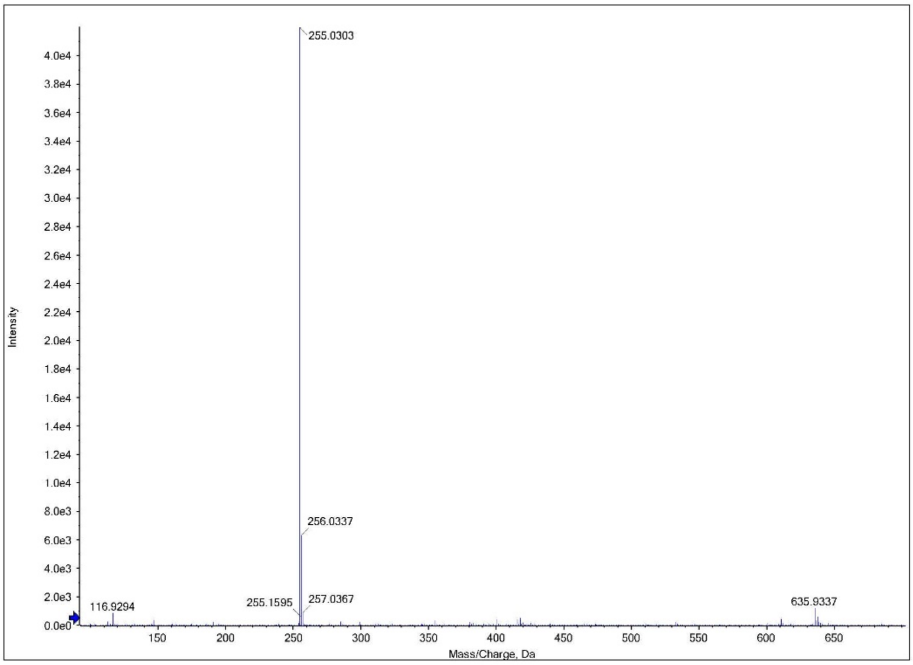

Figure S10. MS spectrum of compound **4**.

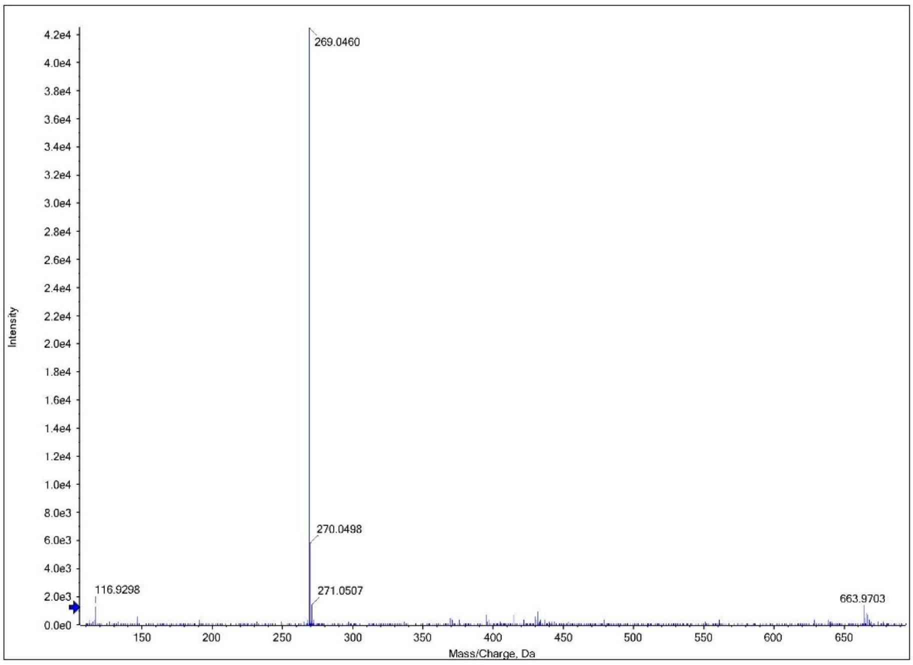

Figure S11. MS spectrum of compound **5**.

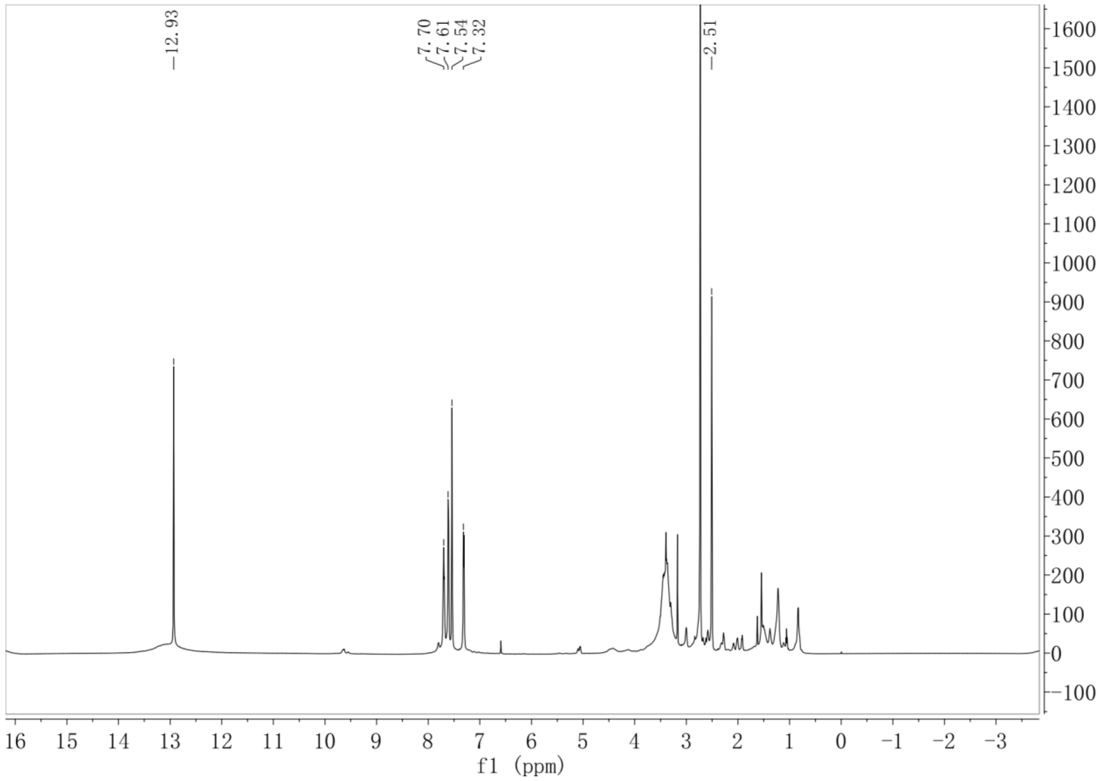

Figure S12. ^{1}H NMR of compound **1**.

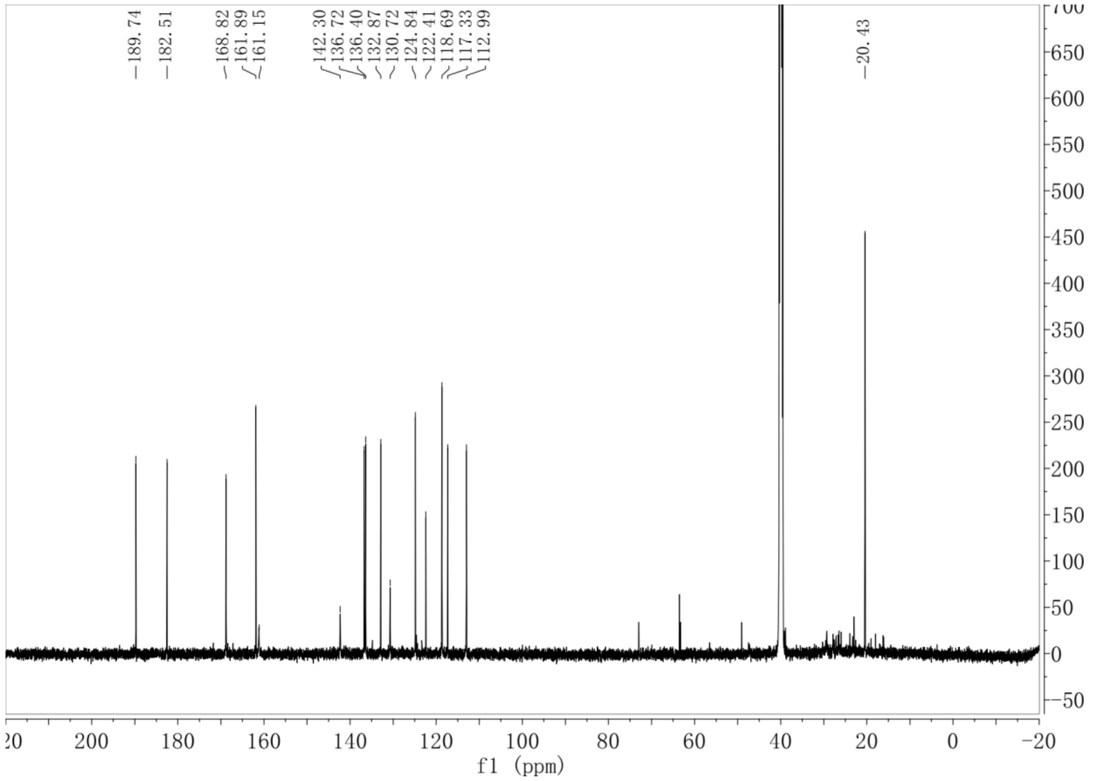

Figure S13. ^{13}C NMR of compound **1**.

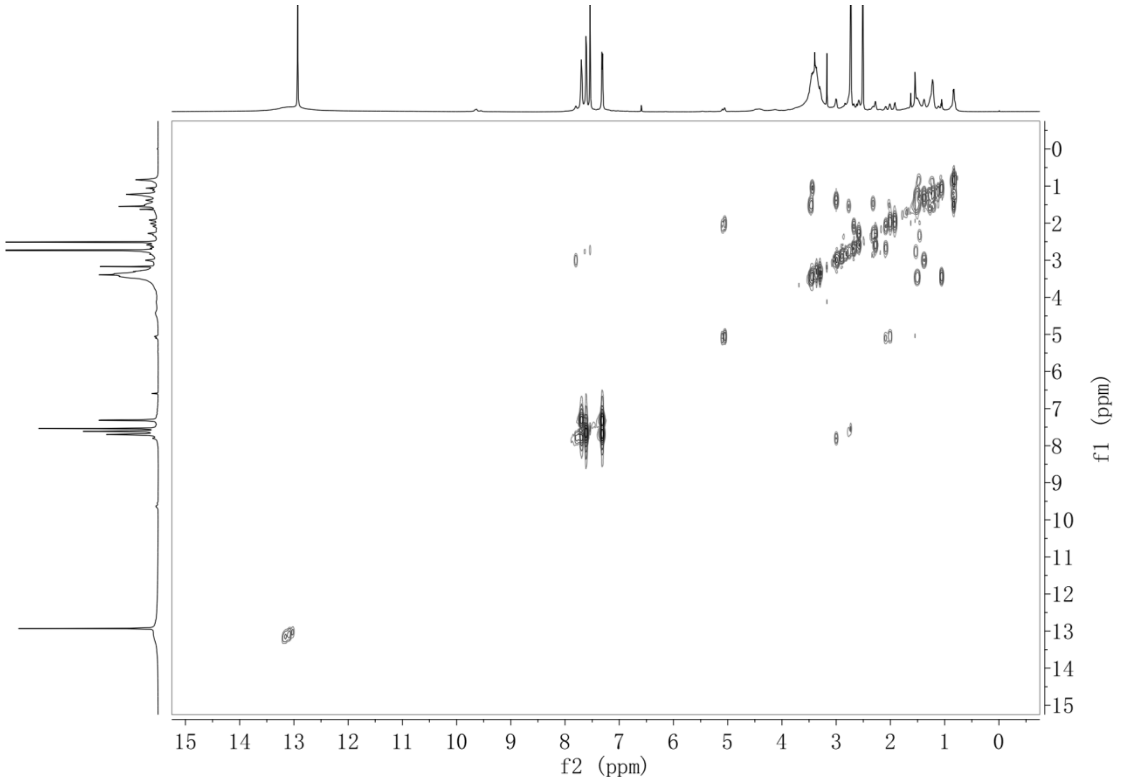

Figure S14. ^{1}H-^{1}H NOESY data of compound **1**.

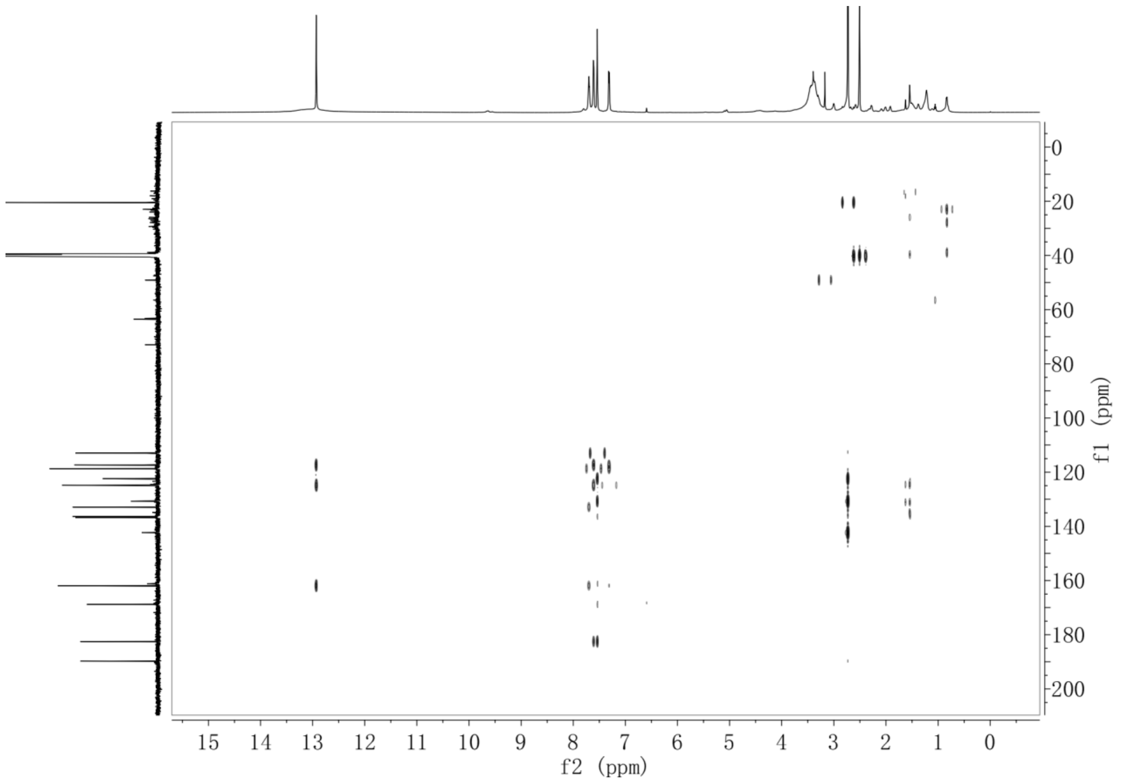

Figure S15. HMQC data of compound **1**.

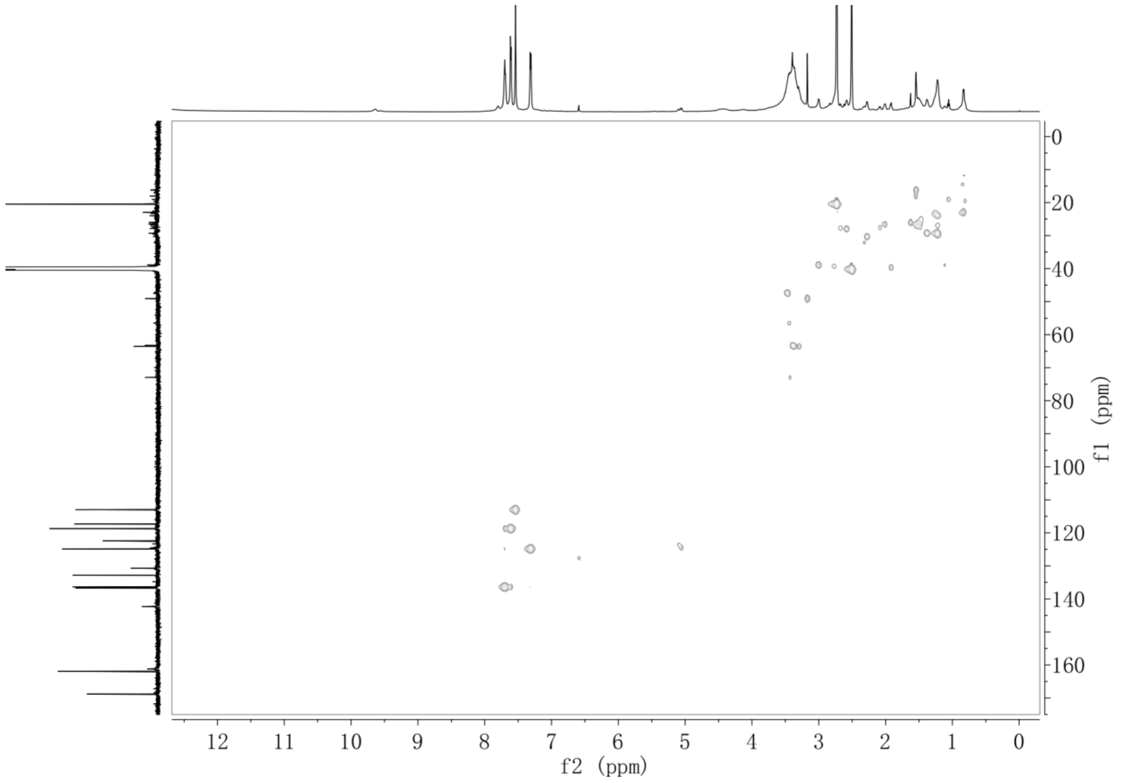

Figure S16. HMBC data of compound **1**.

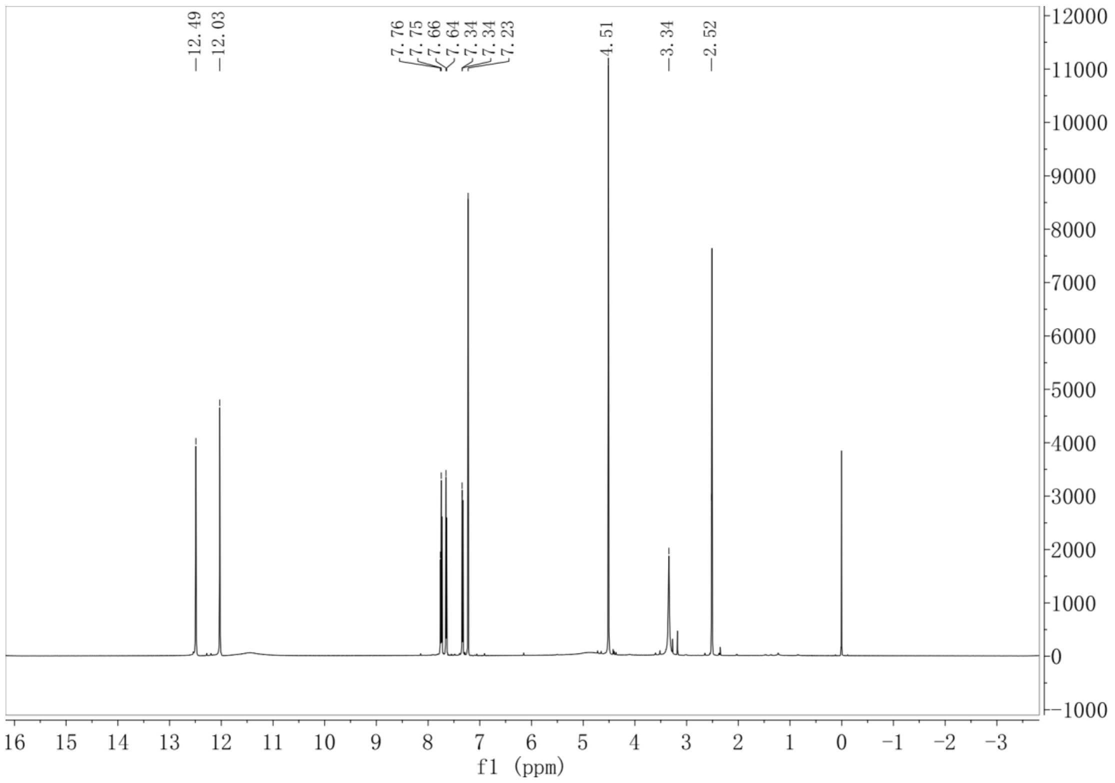

Figure S17. ^{1}H NMR of compound **2**.

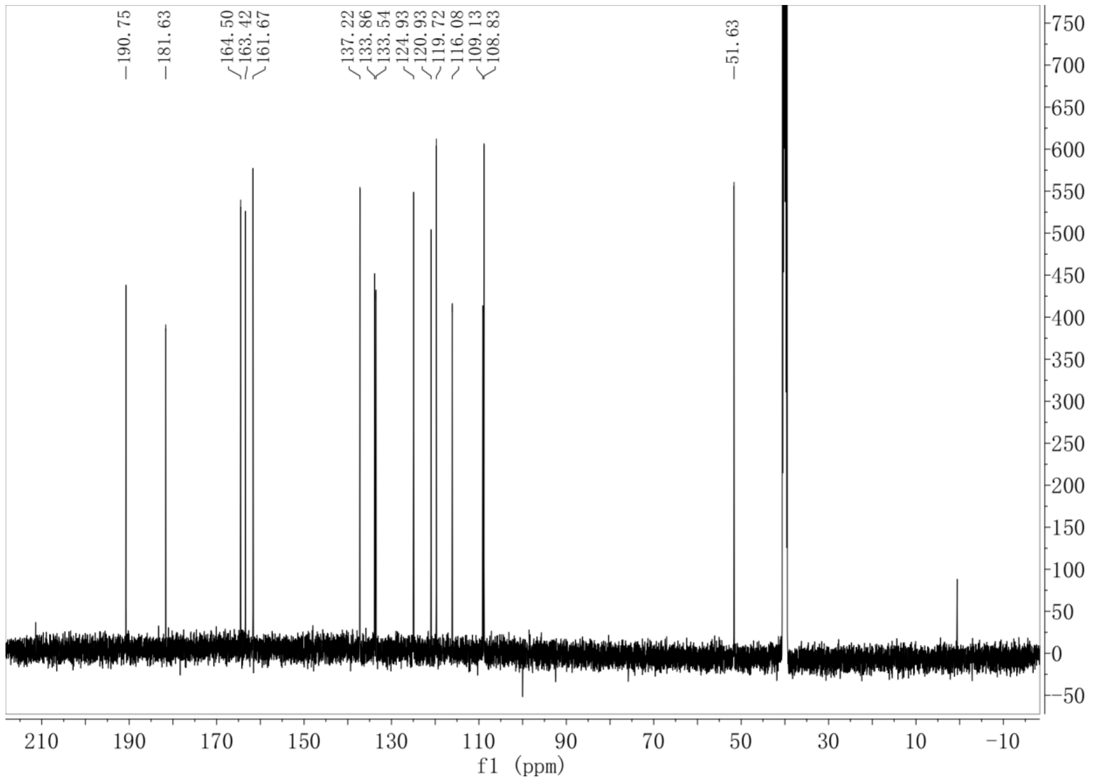

Figure S18. ^{13}C NMR of compound **2**.

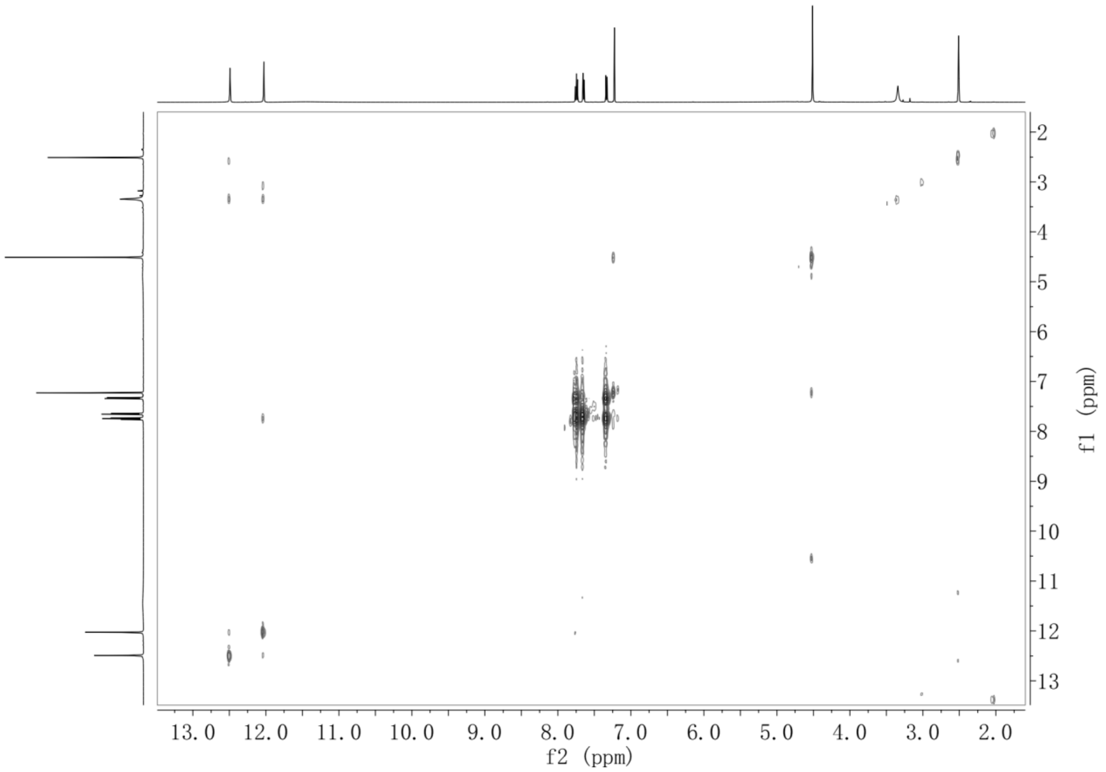

Figure S19. ^{1}H-^{1}H NOESY data of compound **2**.

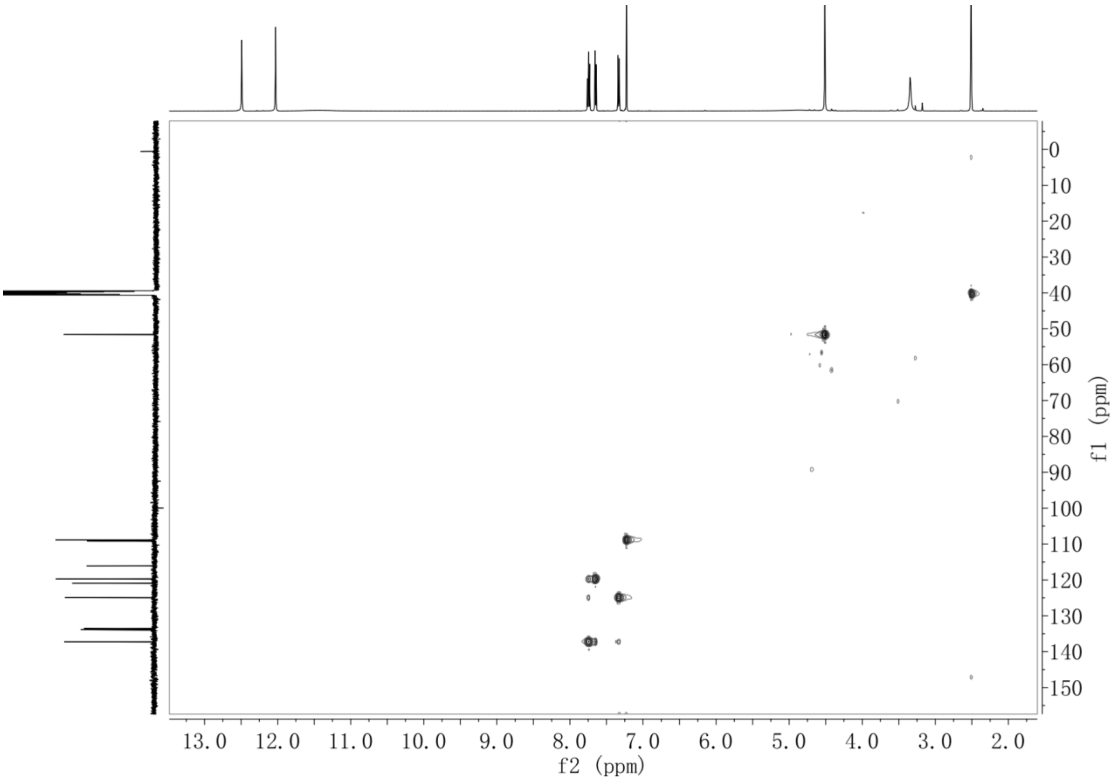

Figure S20. HMQC data of compound **2**.

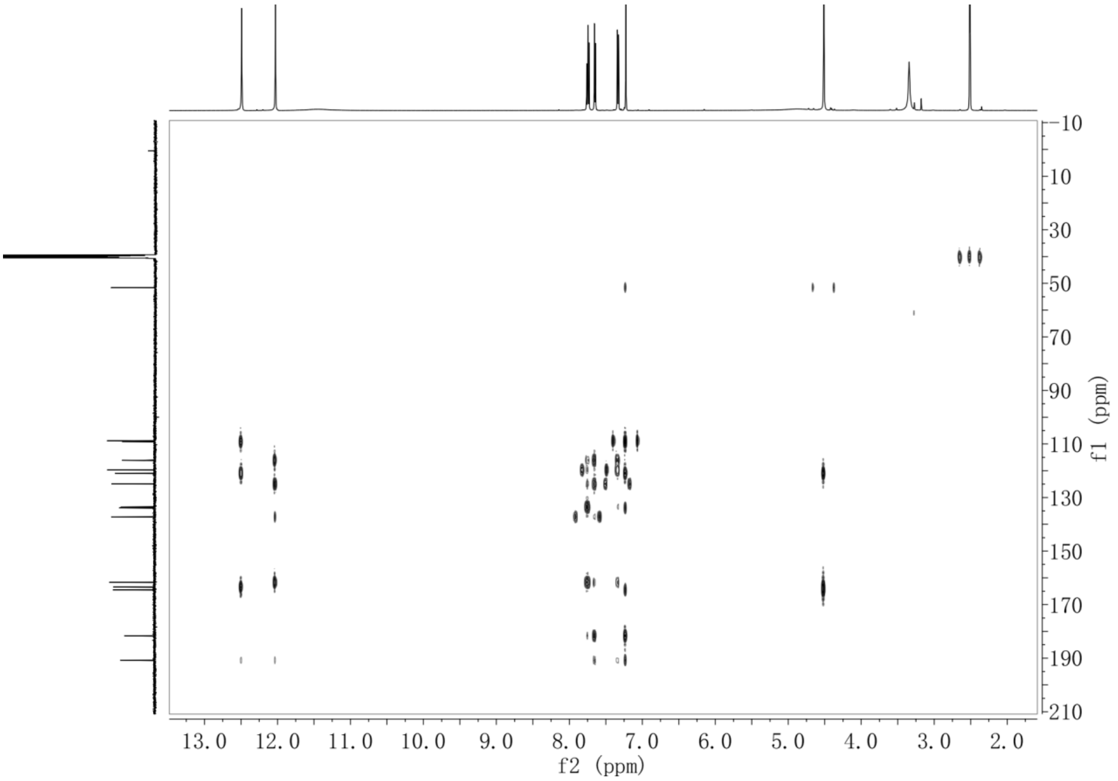

Figure S21. HMBC data of compound **2**.

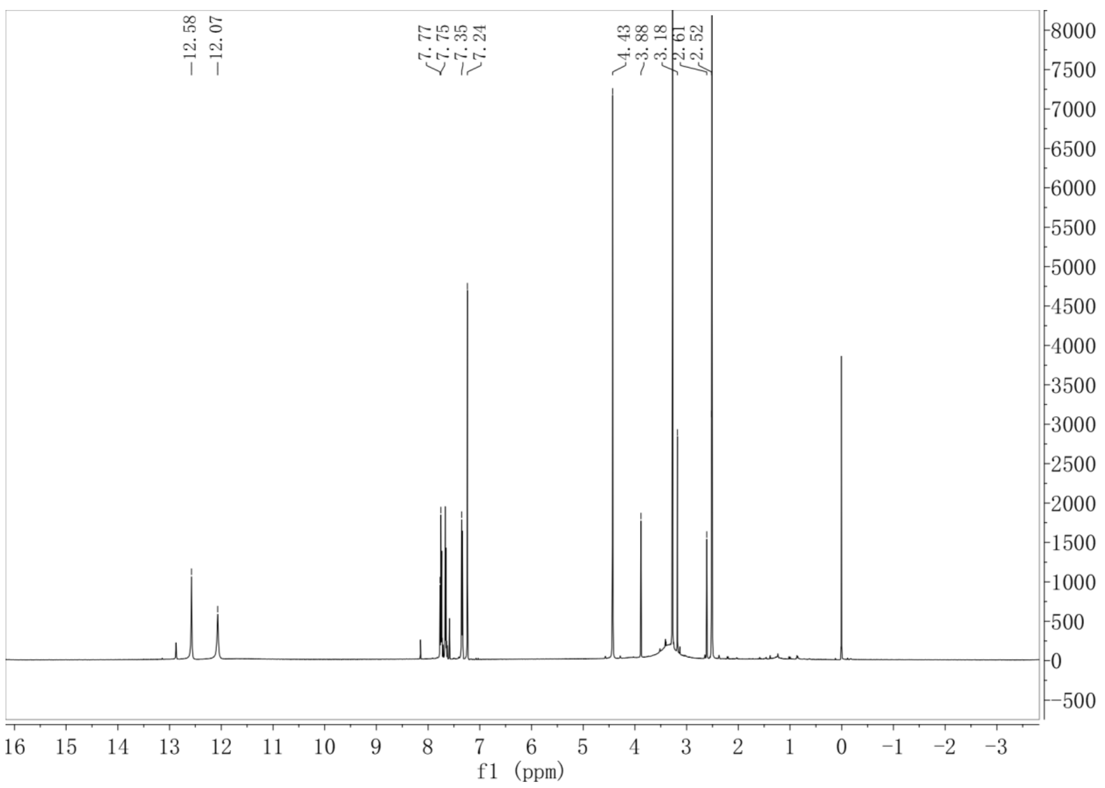

Figure S22. ^{1}H NMR of compound **3**.

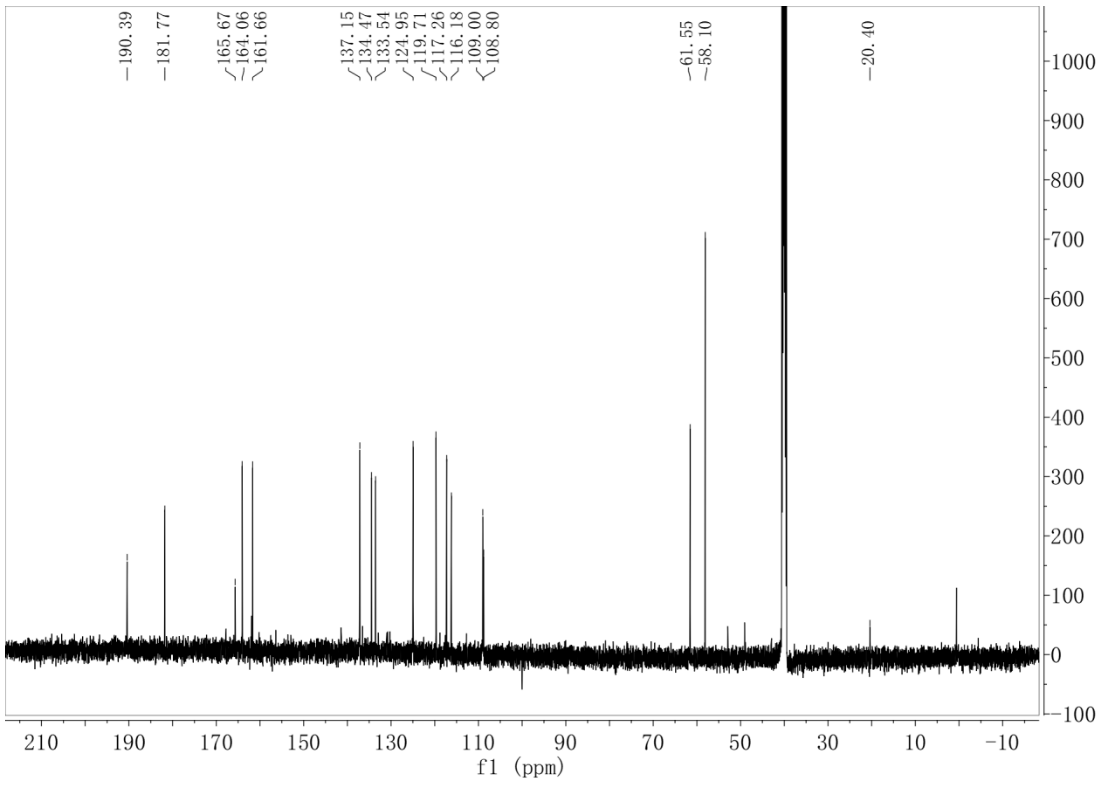

Figure S23. ^{13}C NMR of compound **3**.

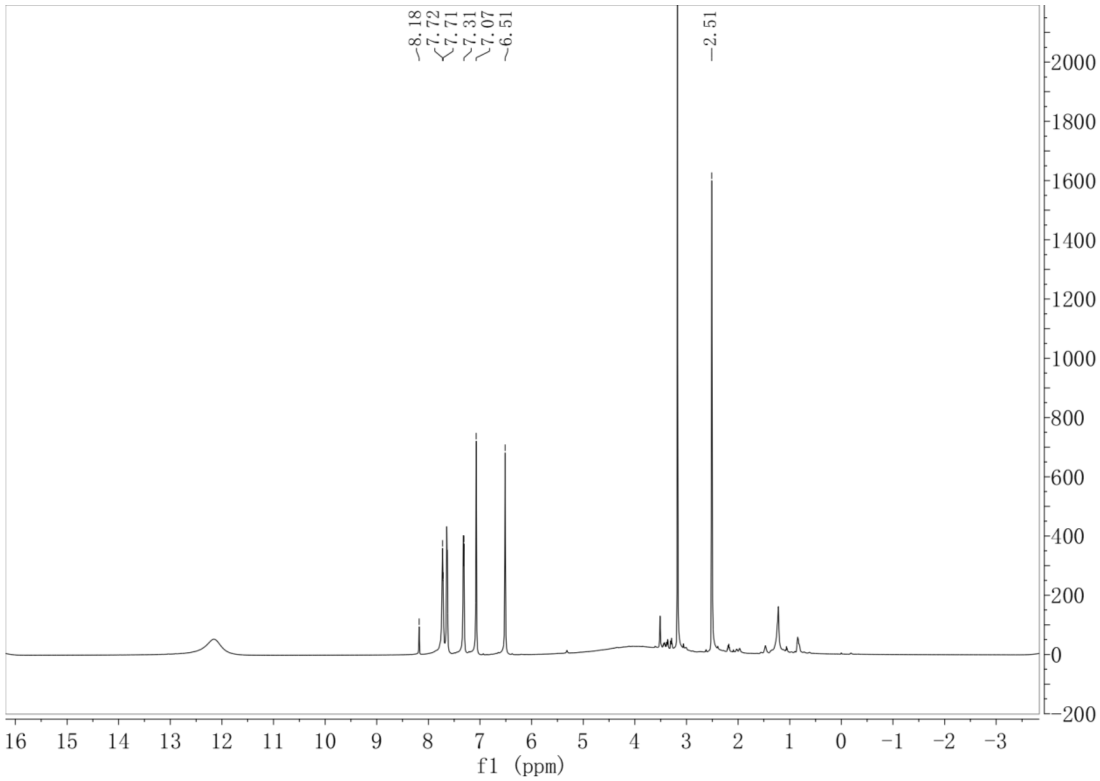

Figure S24. ^{1}H NMR of compound **4**.

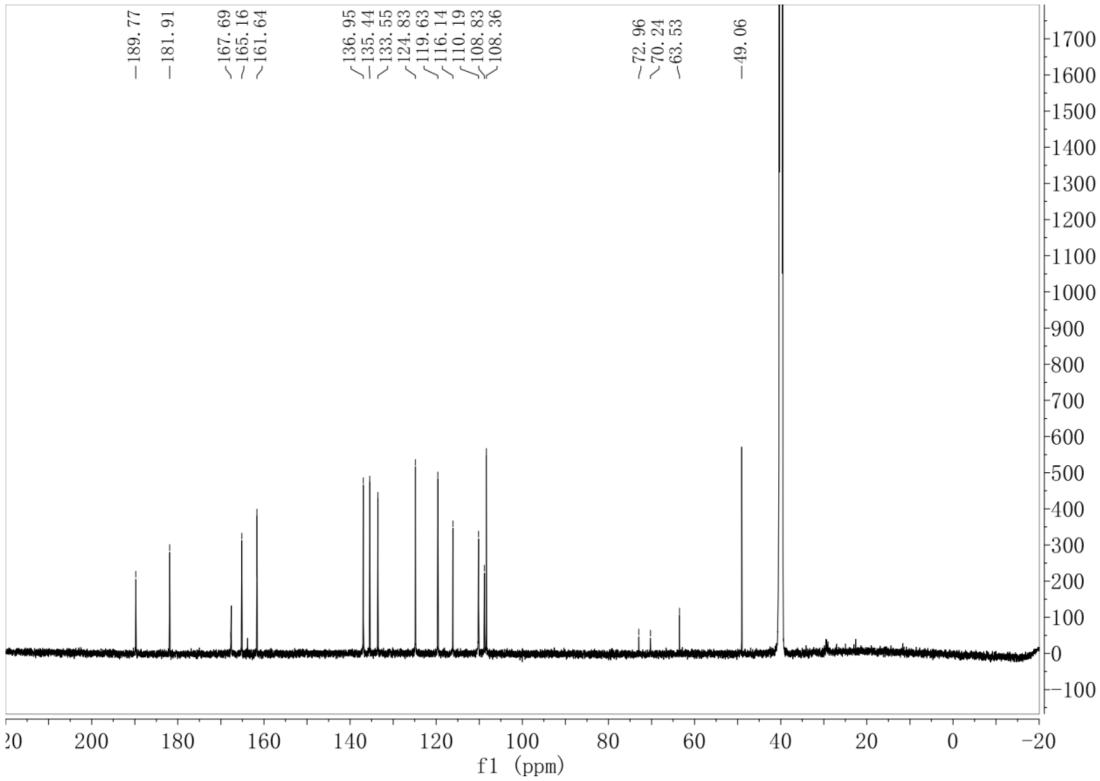

Figure S25. ^{13}C NMR of compound **4**.

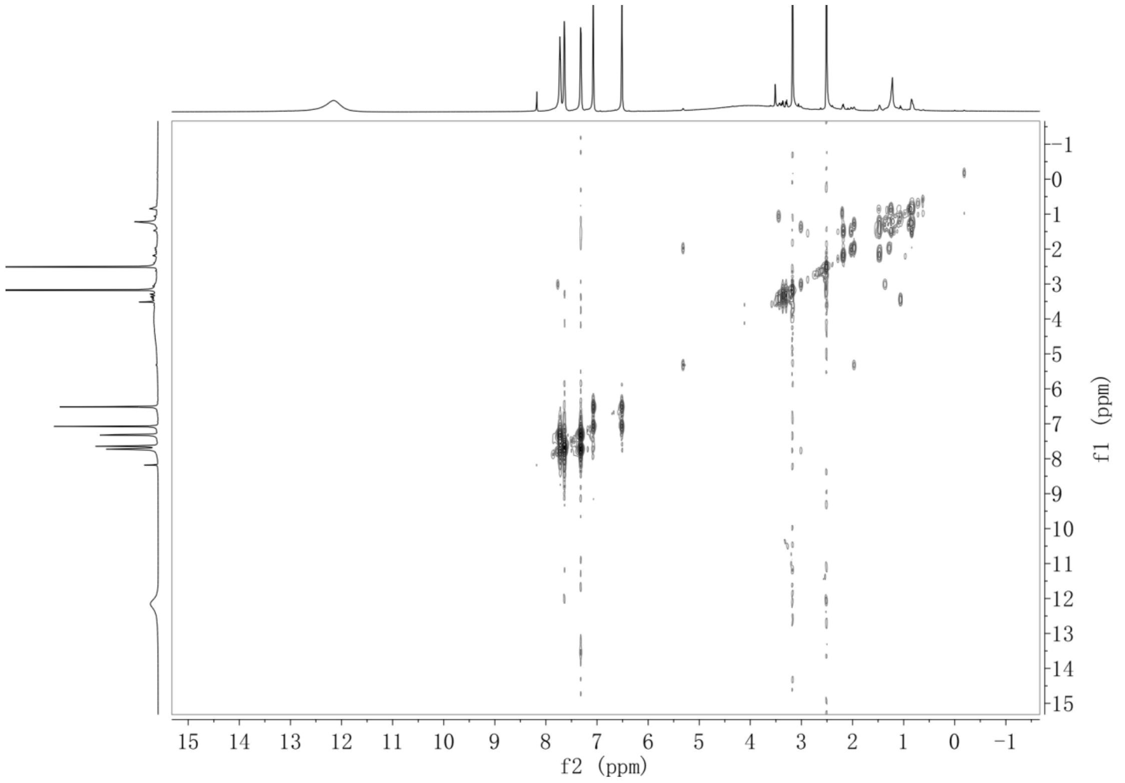

Figure S26. ^{1}H-^{1}H NOESY data of compound **4**.

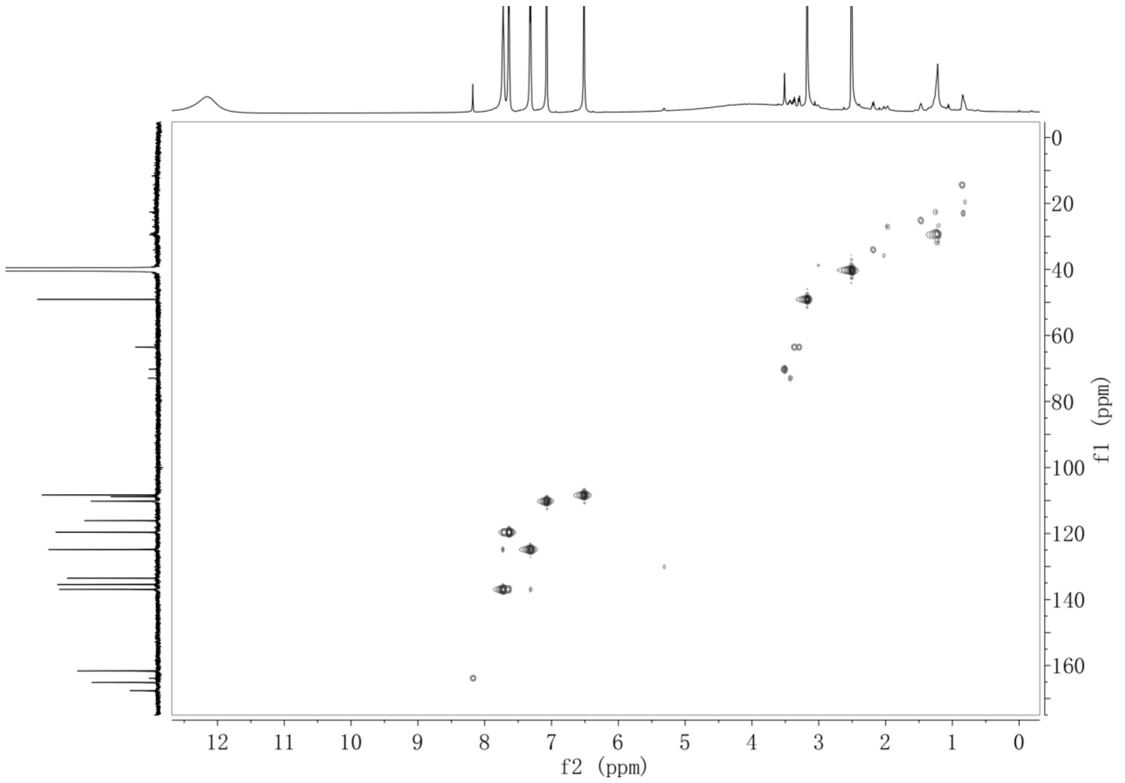

Figure S27. HMQC data of compound **4**.

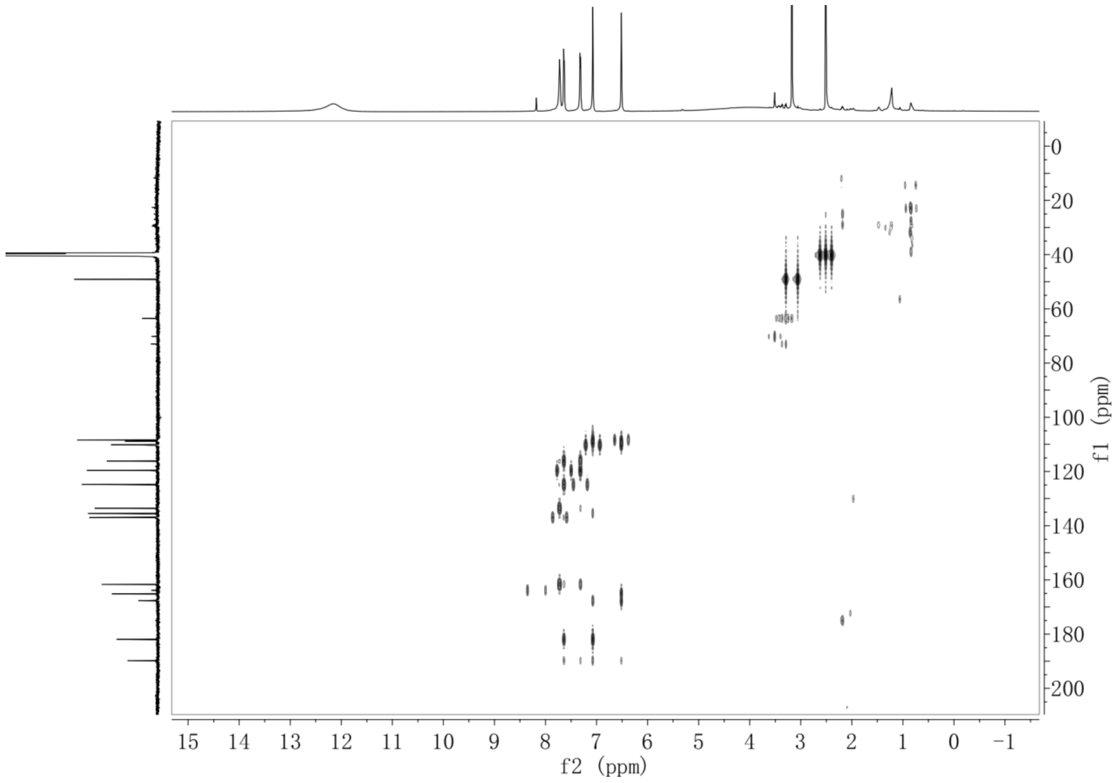

Figure S28. HMBC data of compound **4**.

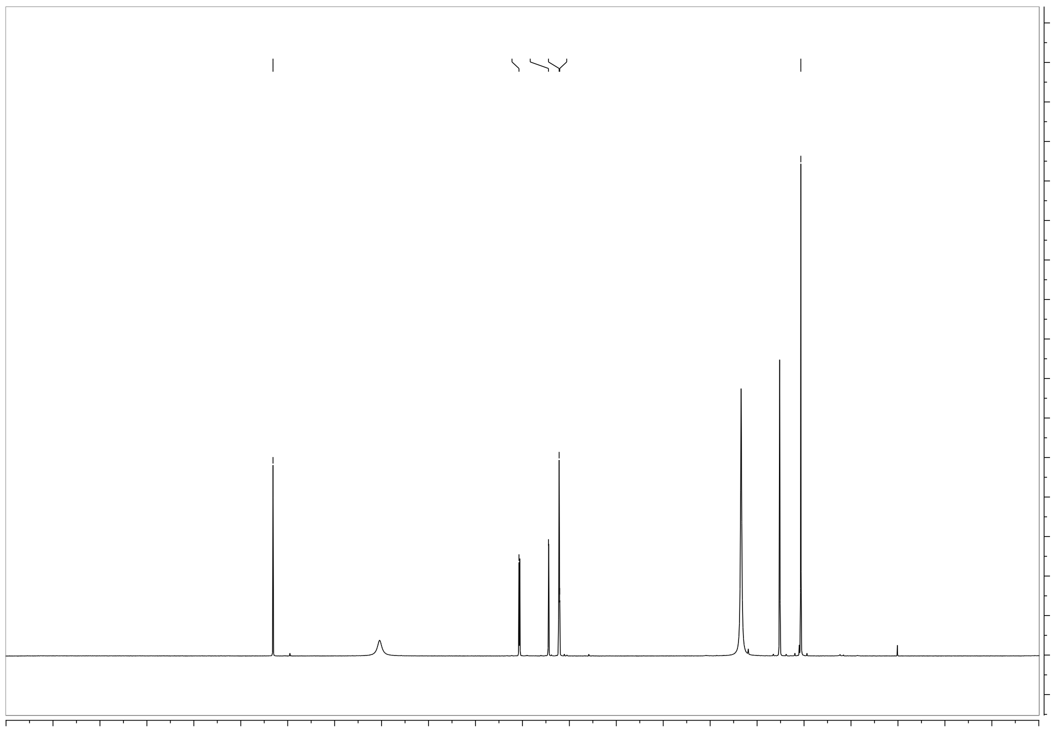

Figure S29. ^{1}H NMR of compound **5**.

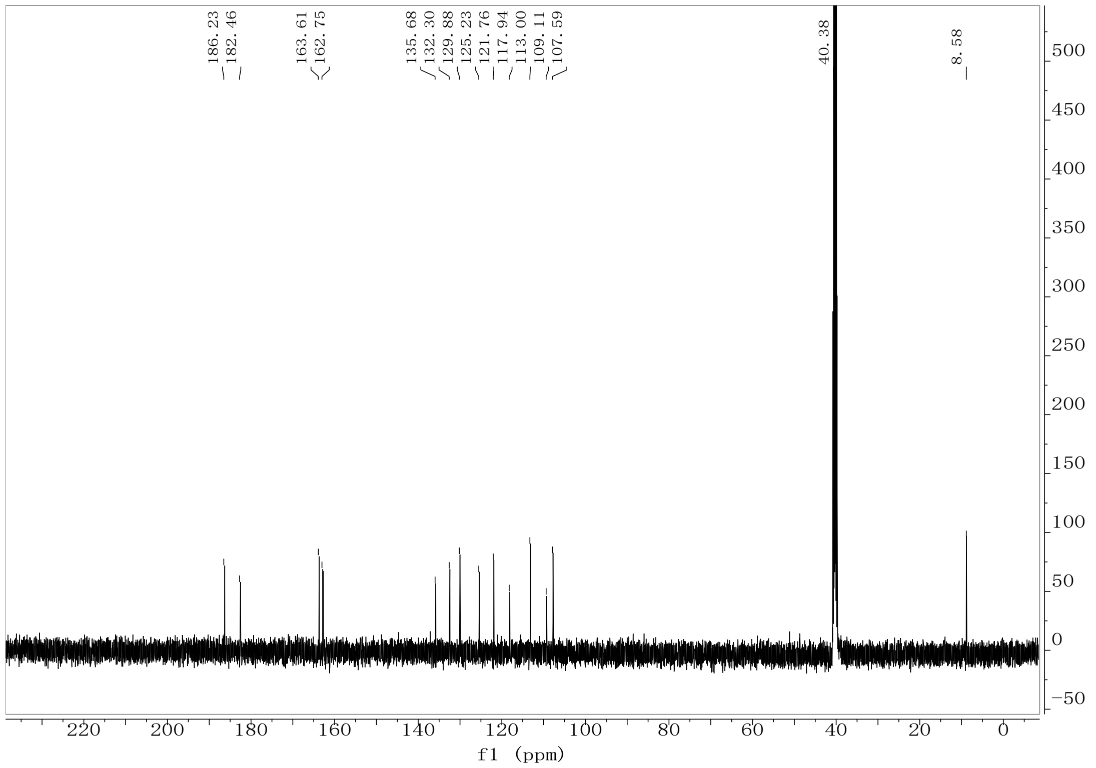

Figure S30. ^{13}C NMR of compound **5**.

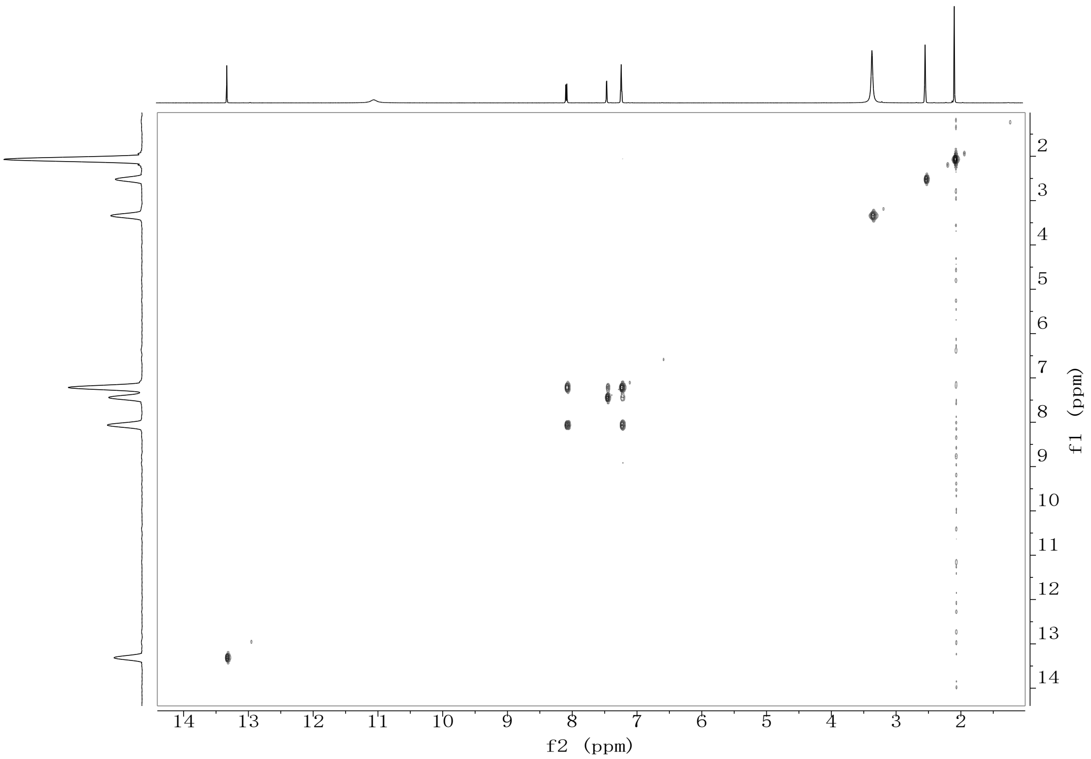

Figure S31. ^{1}H-^{1}H NOESY data of compound **5**.

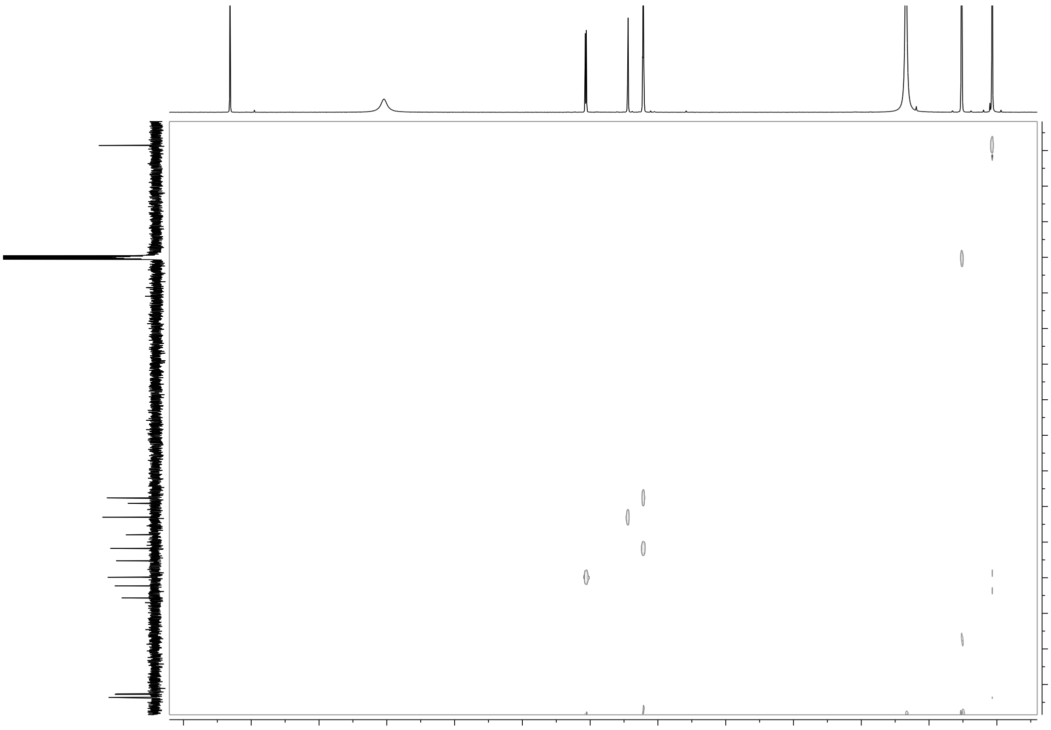

Figure S32. HMQC data of compound **5**.

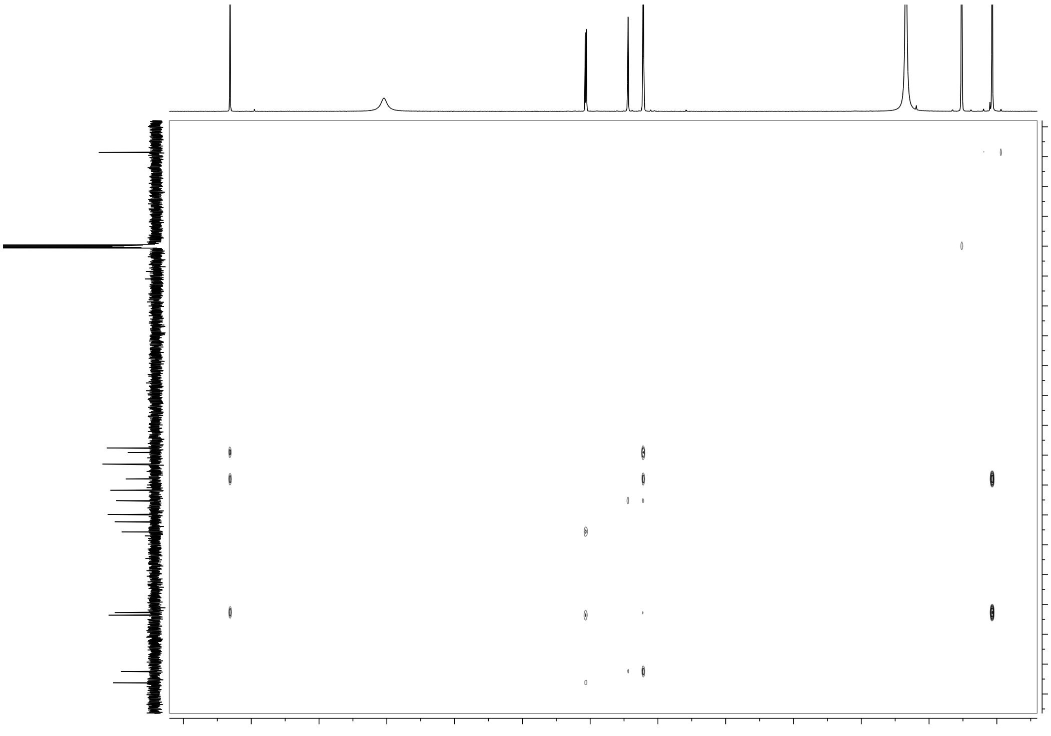

Figure S33. HMBC data of compound **5**.
